# SSNOMBACTER: A collection of scattering-type scanning near-field optical microscopy and atomic force microscopy images of bacterial cells

**DOI:** 10.1093/gigascience/giaa129

**Published:** 2020-11-24

**Authors:** Massimiliano Lucidi, Denis E Tranca, Lorenzo Nichele, Devrim Ünay, George A Stanciu, Paolo Visca, Alina Maria Holban, Radu Hristu, Gabriella Cincotti, Stefan G Stanciu

**Affiliations:** University Roma Tre, Department of Engineering, via Vito Volterra 62, Rome, 00146, Italy; University Politehnica of Bucharest, Center for Microscopy-Microanalysis and Information Processing, 313 Splaiul Independentei, Bucharest,060042, Romania; University Roma Tre, Department of Engineering, via Vito Volterra 62, Rome, 00146, Italy; İzmir Democracy University, Faculty of Engineering, Electrical and Electronics Engineering, 14 Gürsel Aksel Bulvarı, İzmir, 35140, Turkey; University Politehnica of Bucharest, Center for Microscopy-Microanalysis and Information Processing, 313 Splaiul Independentei, Bucharest,060042, Romania; University Roma Tre, Department of Science, via Vito Volterra 62, Rome, 00146, Italy; University of Bucharest, Faculty of Biology, Department of Microbiology and Immunology, 1-3 Aleea Portocalelor, Bucharest, 060101, Romania; University Politehnica of Bucharest, Center for Microscopy-Microanalysis and Information Processing, 313 Splaiul Independentei, Bucharest,060042, Romania; University Roma Tre, Department of Engineering, via Vito Volterra 62, Rome, 00146, Italy; University Politehnica of Bucharest, Center for Microscopy-Microanalysis and Information Processing, 313 Splaiul Independentei, Bucharest,060042, Romania

**Keywords:** scattering-type scanning near-field optical microscopy, atomic force microscopy, bacterial pathogens, dataset, bioimaging

## Abstract

**Background:**

In recent years, a variety of imaging techniques operating at nanoscale resolution have been reported. These techniques have the potential to enrich our understanding of bacterial species relevant to human health, such as antibiotic-resistant pathogens. However, owing to the novelty of these techniques, their use is still confined to addressing very particular applications, and their availability is limited owing to associated costs and required expertise. Among these, scattering-type scanning near field optical microscopy (s-SNOM) has been demonstrated as a powerful tool for exploring important optical properties at nanoscale resolution, depending only on the size of a sharp tip. Despite its huge potential to resolve aspects that cannot be tackled otherwise, the penetration of s-SNOM into the life sciences is still proceeding at a slow pace for the aforementioned reasons.

**Results:**

In this work we introduce SSNOMBACTER, a set of s-SNOM images collected on 15 bacterial species. These come accompanied by registered Atomic Force Microscopy images, which are useful for placing nanoscale optical information in a relevant topographic context.

**Conclusions:**

The proposed dataset aims to augment the popularity of s-SNOM and for accelerating its penetration in life sciences. Furthermore, we consider this dataset to be useful for the development and benchmarking of image analysis tools dedicated to s-SNOM imaging, which are scarce, despite the high need. In this latter context we discuss a series of image processing and analysis applications where SSNOMBACTER could be of help.

## Context

Bacterial pathogens surround us, being found not only in infected patients, but also in soil, water, wild and domestic animals, and food. While diseases caused by some pathogenic bacteria can be prevented by immunization or relieved by antibiotic therapy, others still represent a major public health problem accounting for tens of millions of deaths annually across the globe. Furthermore, some pathogenic species can be regarded as possible warfare agents and thus carry military relevance [1]. Among the most dangerous pathogenic bacterial species in developed countries are those in the ESKAPE group (*Enterococcus faecium, Staphylococcus aureus,Klebsiella pneumoniae, Acinetobacter baumannii, Pseudomonas aeruginosa*, and *Enterobacter* species) [2]. These are among the most common bacterial pathogens in nosocomial infections, causing extensive morbidity and mortality, especially in critically ill and immunocompromised patients [3]. All these species are characterized by a high level of resistance to a variety of antibiotics [4], which recently prompted the World Health Organization to list ESKAPE pathogens among the greatest threats to human health and to boost research on new effective drugs for treatment of antibiotic-resistant infections [5].

A complete and detailed characterization of different bacterial pathogens plays a fundamental role in many biomedical studies, related to bacterial infection diagnosis and treatment. The determination of morphology and other biophysical parameters could provide additional information about both cellular structures and biochemical properties of bacteria. These parameters allow an accurate characterization, which could be used to discriminate pathogenic from harmless commensal bacteria. However, the major part of bacterial structures cannot be investigated in detail by using conventional microscopy techniques owing to resolution limitations. For example, the lateral resolution that can be achieved by using such conventional microscopy techniques based on laser excitation (e.g., confocal laser scanning microscopy) is limited by the light diffraction phenomena to half the wavelength of the excitation light, which translates to a ∼200 nm resolution barrier. As a result, an exact understanding of fundamental structures and processes of bacteria at subcellular levels is yet to be achieved, higher resolution being necessary for elucidating aspects that are still not well comprehended [6, 7]. Optical nanoscopy techniques based on super-resolved fluorescence, such as Stimulated Emission Depletion Microscopy (STED) [8], Fluorescence Photoactivation Localization Microscopy (PALM) [9], or Stochastic Optical Reconstruction Microscopy (STORM) [10] overcome the diffraction barrier, offering typical resolutions in the range of 30–100 nm. However,  their lack of chemical sensitivity and dependence on (very specific) fluorescent probes limits their applicability. In the case of biological samples, the advantages of fluorescence super-resolution microscopy (SRM) techniques come accompanied by a series of drawbacks related to the fact that exogenous and genetically engineered contrast agents can influence the phenotype (e.g., morphology, metabolism, motility) of the cells that are imaged and can also lead to cyto- and phototoxicity. Furthermore, recent studies suggest that unpredictable anomalous processes related to the SRM fluorophore distribution in biological samples exist [11]. These limitations and concerns motivate interest in alternative ways of overcoming the diffraction barrier in the form of optical imaging techniques that do not require contrast agents (label-free).

Among the label-free optical nanoscopy techniques that have emerged over the past years, two prominent families can be easily distinguished: (i) near-field techniques based on the interaction of light and a sharp tip scanned across the sample surface, such as scattering-type scanning near-field optical microscopy (s-SNOM) [12], tip-enhanced fluorescence [13], tip-enhanced photoluminescence [14], tip-enhanced Raman spectroscopy (TERS) [15], photoinduced force microscopy (pi-FM) [16], or photothermal atomic force microscopy [17]; and (ii) far-field techniques based on pump and probe strategies where two or more incident beams compete [18–21]. All these label-free techniques have the capacity to advance the current knowledge on structural, chemical, and optical features of biological samples (and also of advanced [bio]materials). However, due to their novelty, their use is still confined to addressing very specific applications, and their availability is severely limited owing to associated costs and required expertise. Access to datasets collected with these techniques is also widely limited for the same reasons, which translates to huge delays in transferring them to important applications that lie outside the scientific interest of the reduced number of scientific groups developing and using them. Furthermore, modern methods for automated image analysis that have taken the fields of bioimaging (and microscopy in general) by storm over the past few years [22–24] have had insignificant intersections with these emerging label-free modalities, owing to the same reasons expressed above. With this effort, we aim to alleviate the situation by establishing a new trend for sharing relevant datasets collected with such modalities, and other emerging or novel ones. In our view, this would be of great help for enlarging and overcoming the aforementioned bottlenecks.

In the context discussed in the previous paragraph, we focus our attention on s-SNOM, a generally applicable label-free method for surface characterizations at nanoscale resolution [12], whose working principles rely on a sharp tip that is scanned across the sample while being excited with a focused laser beam. The tip converts the incident radiation into a highly localized and enhanced near field at the tip apex, which modifies both the amplitude and the phase of the scattered light. This process depends on the local dielectric properties of the sample [25], given the mutual perturbations occurring between the polarizabilities of the sample and the probe. Interferometric detection of the backscattered light yields thus nanoscale-resolved amplitude and phase images, which can reveal various important properties of nanostructured materials [12, 26]. Measuring the amplitude and the phase changes separately is of interest given that these two different signals contain complementary information about the sample. The amplitude shows the magnitude of the electric field enhancement at the tip apex, which can be quantified by the scattering efficiency, e.g., the number of photons reaching the detector. The phase of near-field signals is related to the complex optical constants using quasi-electrostatic theory [27] and importantly, in their landmark work Stiegler et al. [28] showed that the near-field phase spectra of small particles correlate well with their far-field absorption spectra. Notably, the scattered field comprises a series of terms [29], namely, the incident field (i) scattered by the tip, (ii) scattered by the sample, (iii) scattered by the sample and then by the tip, (iv) scattered by the tip and then by the sample, and (v) scattered by the tip, then the sample, and finally the tip again. The sample properties dictate the weight of each of these terms in the recorded signals, but for an s-SNOM configuration based on detection at higher harmonics of the tip's oscillation frequency, the incident field scattered by the sample should be discarded [29]. From the s-SNOM amplitude and phase images one can typically extract material contrasts instead of absolute values, but in the case of samples where a reference material is available next to unknown ones, absolute values of optical parameters (e.g., refractive index, reflectance) are also available [26].

The complex, but reliable, contrast mechanisms of s-SNOM have thus far enabled a wide range of discoveries in condensed phase materials and 2D materials [30–38]. With respect to imaging biological species, a growing number of experiments demonstrate s-SNOM's usefulness to resolve various properties of biological samples, unavailable with other techniques [39–42]. For example, in the recent work of Mészáros et al. [43], s-SNOM was used to assess the local infrared absorption of single bacterial cells. In particular, illumination at 1,660 cm^−1^ (6,024 nm) was used to visualize the protein content (amide I band) and distribution in a number of representative cells. In the work of Berweger et al. [44], the authors used nano-FTIR spectroscopy (available with an s-SNOM system equipped with a broadband laser) to identify the distribution and density of the membrane protein bacteriorhodopsin in dried purple membrane patches purified from *Halobacterium salinarum*. The authors demonstrated s-SNOM images at 20 nm spatial resolution, with the s-SNOM phase images depicting contrast from the amide I vibrational mode of bacteriorhodopsin available under illumination with 1,667 cm^−1^. In a more recent experiment addressing s-SNOM imaging of the same purple membrane patches of *H. salinarum* based on amide I band contrast, Pfitzner and Heberle [45] introduced a hardware configuration that allows imaging in liquid environments. This represents an especially important feature given that investigating biological samples in their native environment allows various dynamic processes to be assessed including structural changes and others. While in a different experiment the authors used s-SNOM to investigate the same amide bands of individual tobacco mosaic viruses and ferritin complexes, insulin aggregates, and purple membranes [46], another notable effort is that of Paulite et al. [47], who used s-SNOM to assess the composition variations and secondary structure of individual amyloid fibrils, which result from the nucleation-dependent polymerization of proteins, holding important pathological significance wrt. brain disorders. In a different work showcasing the power of s-SNOM with regard to understanding important features of biochemical components, Kästner et al. [48] showed that SNOM-based nano-FTIR spectroscopy can identify and chemically detect domain formation in mixed phospholipid and surfactin monolayers at nanoscale. While these briefly surveyed studies base their conclusions on contrast observable in s-SNOM specific amplitude and phase images/spectra, we find important to mention that s-SNOM amplitude and phase images can be processed to result in maps of the real and imaginary parts of the refractive index (RI). In the previous work of Tranca et al. [40], a proof-of-concept experiment focused on nanoscale RI mapping of erythrocytes showed that such properties (that are much easier to interpret compared with raw s-SNOM data) are easily available with s-SNOM, supposing that a ground-truth reference (a region of known RI) is available on the sample [40]. The utility of s-SNOM imaging for quantitative imaging of the dielectric function and connected optical parameters has been also showcased in a recent experiment in connection to various types of nanomaterials [26].

To augment the popularity of s-SNOM and promote new applications in the life sciences, we introduce here SSNOMBACTER [49], a collection of s-SNOM images assembled by imaging 15 bacterial species, including those in the ESKAPE group. These s-SNOM images come accompanied by registered Atomic Force Microscopy (AFM) data, intrinsically available in an s-SNOM imaging session, owing to the underlying data acquisition principles. The s-SNOM–AFM image pairs are useful for placing nanoscale optical information in a relevant topographic context; the latter's importance for understanding the structure of bacteria is nicely presented in the recent landmark work of Pasquina-Lemonche et al. [50]. The potential uses of the dataset presented here include the topographical, biophysical, and morphological analysis at nanoscale level of different bacterial species. The dataset includes the most representative reference strains of ESKAPE and cystic fibrosis–associated pathogens, including also *Streptococcus pyogenes*, an important human pathogen that causes a wide variety of acute morbidities (soft-tissue infections and pharyngitis), severe life-threatening infections (i.e., streptococcal toxic shock syndrome), and devastating postinfectious sequelae such as rheumatic fever and glomerulonephritis [51]. Notably, the proposed dataset has been assembled by imaging both Gram-positive and Gram-negative bacteria. With respect to the latter, we include in our dataset s-SNOM images collected on an *Escherichia coli* strain, which still represents the most prominent model among Gram-negative bacteria. Given the diversity of information included in the SSNOMBACTER dataset (e.g., optical phase and amplitude, topography, morphology), we believe that it can potentially be useful to devise novel bacterial identification strategies that rely on combined s-SNOM/AFM datasets. For this purpose, additional Gram-positive species were incorporated in SSNOMBACTER, namely, the Gram-positive model organism *Bacillus subtilis* and the commensal/opportunistic pathogen *Staphylococcus epidermidis*. In our view, all tested species could represent a relevant starting point to develop new s-SNOM/AFM image analysis workflows aimed at distinguishing commensal from pathogenic bacteria.

In the following, we describe how the dataset is structured, provide details on how the s-SNOM/AFM imaging was performed, and reflect on a series of computer vision applications where SSNOMBACTER would be useful to support and inspire the development of new s-SNOM–oriented image analysis tools.

## Methods

### Bacterial sample preparation

The bacterial strains used in this work are listed in Table 1. All the bacterial species were routinely grown on nutrient agar plates, except for *S. pyogenes*, which was grown on blood agar plates. Three colonies of each bacterial strain were inoculated in Tryptic Soy Broth or in Todd-Hewitt broth for *S. pyogenes*, and incubated at 37°C for 24 hours under vigorous shaking (300 rpm in an orbital shaker). After the incubation, the bacterial cultures were centrifuged at 3,000*g* × 5 min, washed twice, and diluted in sterile distilled water to reach a final absorption at 600 nm (OD_600_) = 1. Aliquots of 20 μL of each bacterial suspension at OD_600_ = 1 were spotted on glass coverslip (Zeiss, Jena, Germany), with a refractive index of 1.5077 (at 1,550 nm) and air-dried under the laminar flow hood for 20 minutes at room temperature. After the desiccation, the samples were imaged with AFM/s-SNOM.

**Table 1:** Bacterial strains and FOV configurations addressed in the proposed SSNOMBACTER dataset

Bacterial strain	Gram	Bacterial strain reference	No. of imaged regions × FOV dimensions
*Achromobacter xylosoxidans* ATCC 27061 (DSMZ 2402)^T^	−	Yabuuchi and Oyama 1971 [52]	3 × (10 μm × 10 μm); 1 × (2 μm × 2 μm)
*Acinetobacter baumannii* ATCC 17978	−	Sahm et al. 1989 [53]	4 × (10 μm × 10 μm); 1 × (2 μm × 2 μm)
*Acinetobacter baumannii* ATCC 19606^T^	−	ATCC (Bouvet and Grimont 1986) [54]	3 × (10 μm × 10 μm); 2 × (2 μm × 2 μm)
*Bacillus subtilis* subsp. *spizizenii* DSMZ 347	+	ATCC	3 × (10 μm × 10 μm); 1 × (4 μm × 4 μm)
*Burkholderia cenocepacia* ATCC BAA-245 (LMG 16656)^T^	−	Govan et al. 1993 [55]	3 × (10 μm × 10 μm); 1 × (2 μm × 2 μm)
*Enterobacter aerogenes* ATCC 13048 (DSMZ 30053)^T^	−	Bascomb et al. 1971 [56]	3 × (10 μm × 10 μm); 1 × (2 μm × 2 μm)
*Enterobacter cloacae* ATCC 13047 (DSMZ 30054)^T^	−	Hormaeche and Edwards 1960 [57]	3 × (10 μm × 10 μm); 1 × (2 μm × 2 μm)
*Enterococcus faecalis* ATCC 29212	+	ATCC	3 × (10 μm × 10 μm); 3 × (2 μm × 2 μm)
*Enterococcus faecalis* ATCC 700802 (V583)	+	Sahm et al. 1989 [53]	3 × (10 μm × 10 μm); 1 × (2 μm × 2 μm)
*Enterococcus faecium* ATCC 19434 (DSMZ 20477)^T^	+	Schleifer and Kilpper-Bälz 1984 [58]	3 × (10 μm × 10 μm); 1 × (2 μm × 2 μm)
*Escherichia coli* MG1655 (ATCC 700926)^T^	−	ATCC	4 × (10 μm × 10 μm); 1 × (2 μm × 2 μm)
*Klebsiella pneumoniae* ATCC 27736	−	ATCC	3 × (10 μm × 10 μm); 1 × (2 μm × 2 μm)
*Pseudomonas aeruginosa* PAO1 (ATCC 15692)^T^	−	ATCC	3 × (10 μm × 10 μm); 2 × (2 μm × 2 μm)
*Staphylococcus aureus* ATCC 25923	+	ATCC	4 × (10 μm × 10 μm); 1 × (2 μm × 2 μm)
*Staphylococcus aureus* ATCC 43300	+	ATCC	3 × (10 μm × 10 μm); 1 × (2 μm × 2 μm)
*Staphylococcus epidermidis* SP1	+	Spallanzani Hospital, clinical isolate	4 × (10 μm × 10 μm); 1 × (2 μm × 2 μm)
*Stenotrophomonas maltophilia* ATCC 13637 (DSMZ 50170)^T^	−	Palleroni and Bradbury 1993 [59]	4 × (10 μm × 10 μm); 1 × (3 μm × 3 μm)
*Streptococcus pyogenes* ATCC 19615	+	ATCC	4 × (10 μm × 10 μm); 1 × (2 μm × 2 μm)

ATCC: American Type Culture Collection.

At least 3 different 10 × 10 μm fields of view (FOVs) were acquired including both glass substrate regions (used as reference, required for potential quantitative s-SNOM image analyses [26, 40, 60]) and bacterial cells. In addition, ≥1 FOV was imaged at higher magnification (i.e., by scanning a region of lower dimension, namely, of 2 × 2 or 4 × 4 μm). The considered FOV dimensions were selected depending on the dimensions of the selected species; in particular, the minimum FOV has been selected to include a single cell of the species under examination. The image dataset available for each of the considered bacterial species is summarized in Table 1.

### s-SNOM/AFM data acquisition

For acquiring the images available in the SSNOMBACTER dataset we used a NeaSNOM Microscope (Neaspec, Munich, Germany) equipped with a laser source of fixed wavelength, 1,550 nm. Importantly,  s-SNOM configurations are also available with visible, IR, and THz laser excitation sources [61–67], and also with broadband lasers that allow spectroscopic assays [46, 68].

While the wavelength that we used (1,550 nm = 6,451.61 cm^–1^) was not specifically chosen to match a particular optical property of the bacteria (e.g., the absorption properties of bacterial components lie elsewhere [69]), it nonetheless provides s-SNOM amplitude and phase images that correspond to the dielectric properties of the investigated sample, as discussed earlier. Furthermore, it is important to mention that despite the bacterial samples having been desiccated before imaging with AFM/s-SNOM, a small amount of water may have been however retained by these in response to the osmotic shock induced by dehydration [70]. Thus, some features in the s-SNOM phase images may be linked to the absorption of water molecules near/on the bacterial membrane. One additional important aspect to discuss is the non-invasive effect of the wavelength used. In a previous work [71], the effects of lasers with wavelengths ranging from 500 to 1,550 nm were investigated on different bacterial species (i.e., the Gram-negative *E. coli* and the 3 Gram-positive microorganisms *B. subtilis, Bacillus cereus*, and *Micrococcus luteus*), 2 of which are included in the database presented in our work (i.e., *E. coli* and *B. subtilis*). The 1,550 nm laser line showed the lowest laser-induced cell lysis, preserving the intracellular RNA and minimizing the effects on intracellular enzymatic activity. Thus, the features available in the s-SNOM phase and amplitude images correspond to the intrinsic properties of desiccated bacteria (except for potential water contamination) and not to potential compounds that may result from phototoxicity.

In s-SNOM the available resolution is dictated by the size and geometry of the tip, and in the case of this experiment a Hq: NSC19/Cr-Au gold-coated probe (Mikromasch, Sofia, Bulgaria) with <35 nm tip radius was used. Its resonance frequency and force constant are 65 kHz and 0.5 N/m, respectively.

## Dataset Structure

SSNOMBACTER is a dataset comprising 4,400 images collected with AFM and s-SNOM in various workmodes, each of these made available in both .tiff and .gsf file format. The .tiff files can be opened with any image viewer/processing software, e.g., the freeware image viewer IrfanView or ImageJ, while the .gsf files represent the default file format of the NeaSNOM system that was used in this experiment for AFM/s-SNOM imaging. The .gsf files can be accessed with the open-source Gwyddion software [72]. The collection of .tiff and .gsf files is divided in 15 folders, 1 for each of the bacterial species reported in Table 1. Some of the folders are further structured in subfolders, depending on the bacterial strains, which exist for some of the considered species. Each bacterial strain (or bacterial species) folder harbors a number of subfolders that are numerically titled. For each sample ≥3 FOVs of 10 × 10 μm were imaged; for this FOV dimension the names of these subfolders are equivalent to the number of the imaged sample region. For lower dimension FOVs (e.g., 2 × 2 or 4 × 4 μm), the FOV dimension (and FOV number) are explicitly presented in the subfolder name. The number of imaged FOVs for each specimen is presented in Table 1. For each FOV we provide data collected in complementary s-SNOM and AFM imaging modes, listed in Table 2.

**Table 2:** List of abbreviations of the AFM/s-SNOM imaging modes

Abbreviation **^a^**	Description
M0A	AFM topography error
M1A	AFM topography error; first harmonic
M2A	AFM topography error; second harmonic
M3A	AFM topography error; third harmonic
M4A	AFM topography error; fourth harmonic
M5A	AFM topography error; fifth harmonic
M1P	AFM topography phase; first harmonic
M2P	AFM topography phase; second harmonic
M3P	AFM topography phase; third harmonic
M4P	AFM topography phase; fourth harmonic
M5P	AFM topography phase; fifth harmonic
O0A	s-SNOM amplitude
O1A	s-SNOM amplitude, first harmonic
O2A	s-SNOM amplitude, second harmonic
O3A	s-SNOM amplitude, third harmonic
O4A	s-SNOM amplitude, fourth harmonic
O5A	s-SNOM amplitude, fifth harmonic
O0P	s-SNOM phase
O1P	s-SNOM phase, first harmonic
O2P	s-SNOM phase, second harmonic
O3P	s-SNOM phase, third harmonic
O4P	s-SNOM phase, fourth harmonic
O5P	s-SNOM phase, fifth harmonic
Z	AFM Topography

a The letter R reported in the dataset files indicates that the image was collected in the reverse scanning direction (e.g., RZ—topography collected in reverse scanning direction).

The *.gsf and the corresponding *.tiff are titled according to the following nomenclature:

[bacterial strain]_[FOV_number] [imaging_mode_abbreviation].

**Figure 1:**
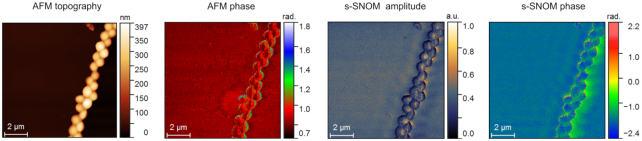
Sample AFM and s-SNOM images collected on *S. aureus* ATCC 25923. The AFM phase image corresponds to the first harmonic of the tip's tapping frequency (M1P); the s-SNOM amplitude and phase images correspond to the third harmonic of the tip's tapping frequency (O3A, O3P).

For instance, the filename “*Achromobacter xylosoxidans* ATCC 27061_1 M2A.gsf” indicates that the respective *.gsf file corresponds to the second harmonic of the topography error image, collected on the first FOV for *A. xylosoxidans* strain ATCC 27061. In each FOV folder we provide a *.txt file that presents all the acquisition parameters used for the respective measurement (e.g., pixel area, scan area). In Fig. 1, we provide a sample image subset consisting in AFM topography and phase, and s-SNOM amplitude and phase images collected on *S. aureus* ATCC 25923.

Each file of SSNOMBACTER [49] can be downloaded individually, or the whole set can be downloaded as a ZIP archive.

## Reuse: utility of SSNOMBACTER for the development of s-SNOM–oriented computer vision applications

The SSNOMBACTER dataset consists of 4,400 images collected with s-SNOM and AFM modalities on 15 bacterial species. The dimensions of this dataset can be further expanded by processing the available images to extract other types of data representations (e.g., by assembling 3D representations from the available AFM topographic information or by calculating dielectric function maps from the amplitude and phase s-SNOM images [26]). A different way to expand our dataset can rely on data augmentation strategies that apply various transformations to an initial image in order to render new representations that simulate other potential acquisition conditions. Such data augmentation strategies have been demonstrated as being particularly useful in deep learning approaches [73]. Given the content, dimension, and variability available in our dataset, we envision that it represents a useful resource to develop novel image processing and analysis tools dedicated to AFM and s-SNOM imaging, and benchmark existing ones. While such tools have already been reported for AFM imaging, they are still largely unavailable for the more recent s-SNOM modality, whose spread and number of applications have escalated over the past years [12]. The importance of reference datasets that enable objective comparisons between competing microscopy-oriented image analysis/processing approaches is discussed in detail by Rubens et al. [74]. In the following, we discuss potential use-cases of our dataset.

### Image restoration and denoising

By employing digital restoration methods, an image whose quality is affected by noise, artifacts, or improper acquisition conditions is processed to obtain a better estimate of the original object. The aforementioned causes are technique or equipment dependent; thus they greatly differ between imaging modalities that rely on optical and scanning probe principles. In the case of the latter, probe damage, mismatch between probe and sample geometry, scanner drift, vibrations, surface contamination, and others impede an unbiased visualization of the imaged sample [75, 76]. Furthermore, these causes are further extended in s-SNOM by inconsistencies in the alignment of the s-SNOM excitation beam and the apex of the probe, which may occur during image collection [77]. Such inconsistencies translate to signal variations that raise problems with respect to manual and automated analysis of the recorded image. Moreover, interferences between near-field and background signals contained in the scattered field contributing to the image are also known to produce artifacts in s-SNOM [78].

**Figure 2:**
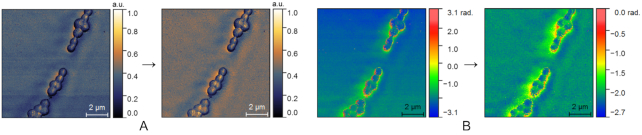
Restoration of s-SNOM data by digital image processing. The raw s-SNOM amplitude (A) and phase image (B) collected on *S. aureus* ATCC 25923 have been processed in the Gwyddion software with 3 operations: “Align rows by median,” “Correction of horizontal strokes,” and “Correct small grains marked by >90% threshold by interpolation.” The resulting s-SNOM images (right of arrow) have homogeneous background, and the bacterial cells are displayed with better contrast.

The proposed dataset can be used to develop novel image restoration methods oriented towards s-SNOM and AFM imaging and benchmark existing ones. We envision 2 potential scenarios for such efforts. In the first, the images available in our set can be restored (Fig. 2), and afterwards the quality of the corrected image can be evaluated by means of no-reference (blind) image quality algorithms [79]. In an alternative approach, the proposed images can be regarded as ground truth, and degraded instances can be synthetically generated. In this second case, because ground truth exists, the results of image restoration methods developed for addressing AFM and s-SNOM data can be evaluated by means of full-reference image quality assessment algorithms [80].

### Quantitative imaging meets image fusion and correlative display

**Figure 3:**
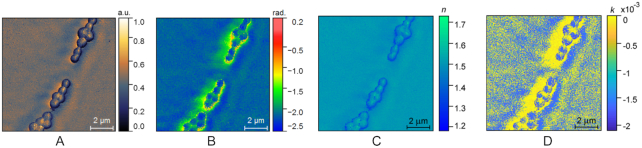
Quantitative representation of the refractive index (imaginary part and real part) assembled using s-SNOM amplitude and phase images collected on *S. aureus* ATCC 25923 under different settings, using a previously reported methodology [40]. (A) s-SNOM amplitude (O3A); (B) s-SNOM phase (O3P); (C) refractive index real part (n); (D) refractive index imaginary part (k) (a.k.a. extinction coefficient).

Quantitative imaging is especially important for achieving an in-depth understanding of both biological and materials samples. The availability of quantifiable features allows objective analyses to be performed on the sample properties, and consequently unbiased conclusions to be drawn. While topographic information collected with AFM is intrinsically quantitative in nature, Tranca et al. demonstrated that s-SNOM images of the same FOV, but collected under different acquisition conditions (e.g., different modulation harmonics) or depicting complementary information (amplitude and phase), can be processed on the basis of a methodology that relies on the oscillating point dipole model, in order to extract a nanoscale map of precise values of the dielectric function, and intrinsic optical properties (e.g., refractive index, absorption, reflectance) [40, 60] (Fig. 3). In a follow-up study [26], the usefulness of such approaches was demonstrated in the case of different types of distinct nanomaterials. The availability of AFM and s-SNOM images enables the development and benchmarking of methods aimed at fusing and integrating these complementary information categories, which is useful for their joint visualization and analysis. The importance of software tools that jointly process and display topographic and optical data has been thoroughly discussed [81–84].

### Image segmentation

**Figure 4:**
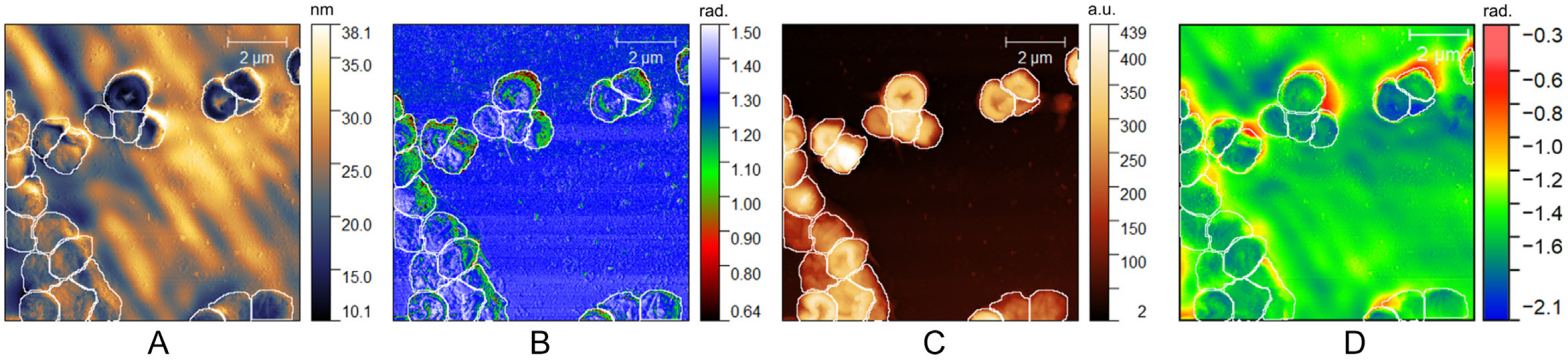
Multi-modality segmentation example of amplitude and phase images of s-SNOM and AFM collected on *Acinetobacter baumannii* ATCC 17978. The boundaries of the bacteria present in the field of view are manually delineated and visualized as overlays using the publicly available ImageJ/FIJI program. (A) AFM amplitude; (B) AFM phase; (C) s-SNOM amplitude; (D) s-SNOM phase.

Image segmentation is the process of partitioning an image into sets of pixels known as segments to allow for easier and meaningful representation [85]. In bioimage analysis applications, image segmentation is a crucial task, which precedes further analyses carried out at the single structure (e.g., cell) level [86, 87]. Specific to the dataset proposed here, segmentation of bacteria can be realized by using images corresponding to a single or to multiple modalities. In the latter scenario, complementary information originating from entirely distinct modalities (e.g., AFM and s-SNOM) or from distinct but related contrasts of the same modality (e.g., amplitude and phase images of s-SNOM) can be used to design segmentation algorithms with improved segmentation accuracy (Fig. 4). The availability of images of bacteria collected with different techniques or different contrast principles of the same techniques is also useful to support the development and benchmarking of generic algorithms that aim to be workmode invariant [88, 89]. Furthermore, it can support the development of adversarial methods that transfer knowledge [90, 91] learnt from a widely available imaging modality (e.g., confocal or brightfield) to 1 or more imaging modalities with reduced availability (e.g., s-SNOM) for which sufficient labeled training data are not available. SSNOMBACTER can thus help expand past work that has been done on segmenting bacteria, addressing important tasks such as classification [92], proliferation and lineage analyses [93], and others [87, 94].

### Image feature extraction

**Figure 5:**
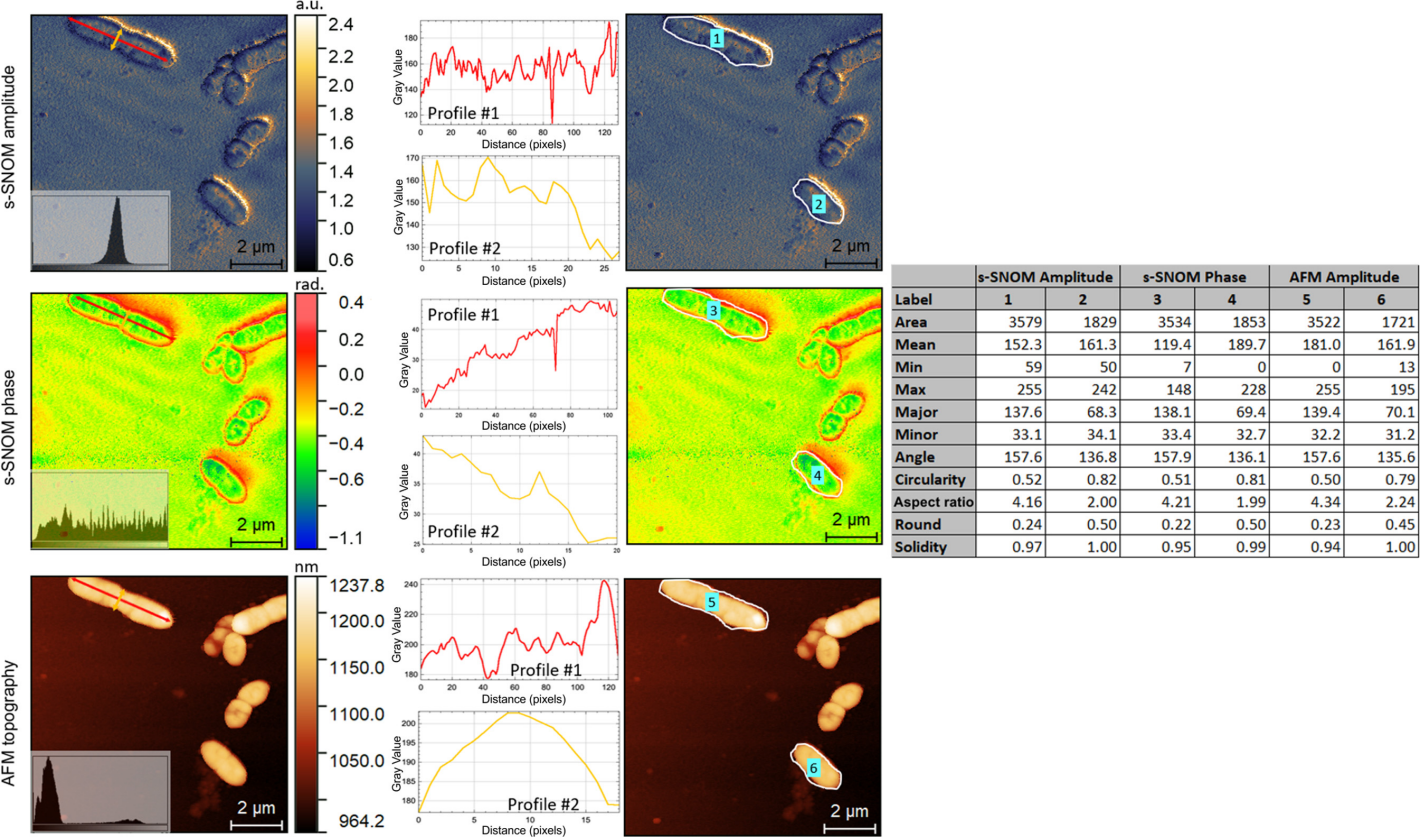
Example on automated feature extraction in the case of s-SNOM amplitude and phase and AFM topography images collected on *Burkholderia cenocepacia* ATCC BAA-245 using an in-house–developed software. Once the user clicks on a bacteria, ellipse fitting on the gradient image is realized and the intensity profiles along the major and minor axes of the fitted ellipse are extracted and displayed along with various features such as area and circularity of the ellipse.

Previous studies have shown that morphological features such as shape, cell size and size distribution, cell wall thickness, and many others can be useful in distinguishing between various bacterial species or between different types of the same species [95]. Measuring such properties manually is possible but tedious and time consuming. Fortunately, computer vision algorithms can be of great help for automating such tasks, but obviously they need to be developed and benchmarked using relevant datasets. SSNOMBACTER comprises both AFM and s-SNOM images of 15 bacterial species, collected at different scales, and hence can consistently support such efforts. The provided image sets are helpful in developing methods that automatically identify and extract various descriptive features, whose importance for various tasks has already been demonstrated, or for designing new features that can bring added value to important problems such as diagnostics, screening, and so forth. Furthermore, the proposed dataset can also support the development of methods that exploit such descriptive features to answer various biologically motivated image analysis questions, such as “can we distinguish one bacterial species from the others by applying machine learning on images?” or “can we discriminate viable bacteria from the dead using image features only?” Such methods for automated classification/identification of different bacterial types can obviously be of immense help for saving time and human resources [96]. Notably, given that the s-SNOM and AFM images are by default registered, SSNOMBACTER supports as well the development of computer vision algorithms capable to generalize, and hence to address dual-mode or multi-mode imaging applications [97]. Fig. 5 displays an exemplary use-case on identification of image features extracted from bacteria on AFM and s-SNOM data, where information such as intensity profiles or size- and shape-related characteristics (e.g., area, roundness) from bacteria regions can be obtained. As aforementioned, such features can subsequently be used in various computer vision tasks promoting speed and efficiency.

### Image registration and stitching

**Figure 6:**
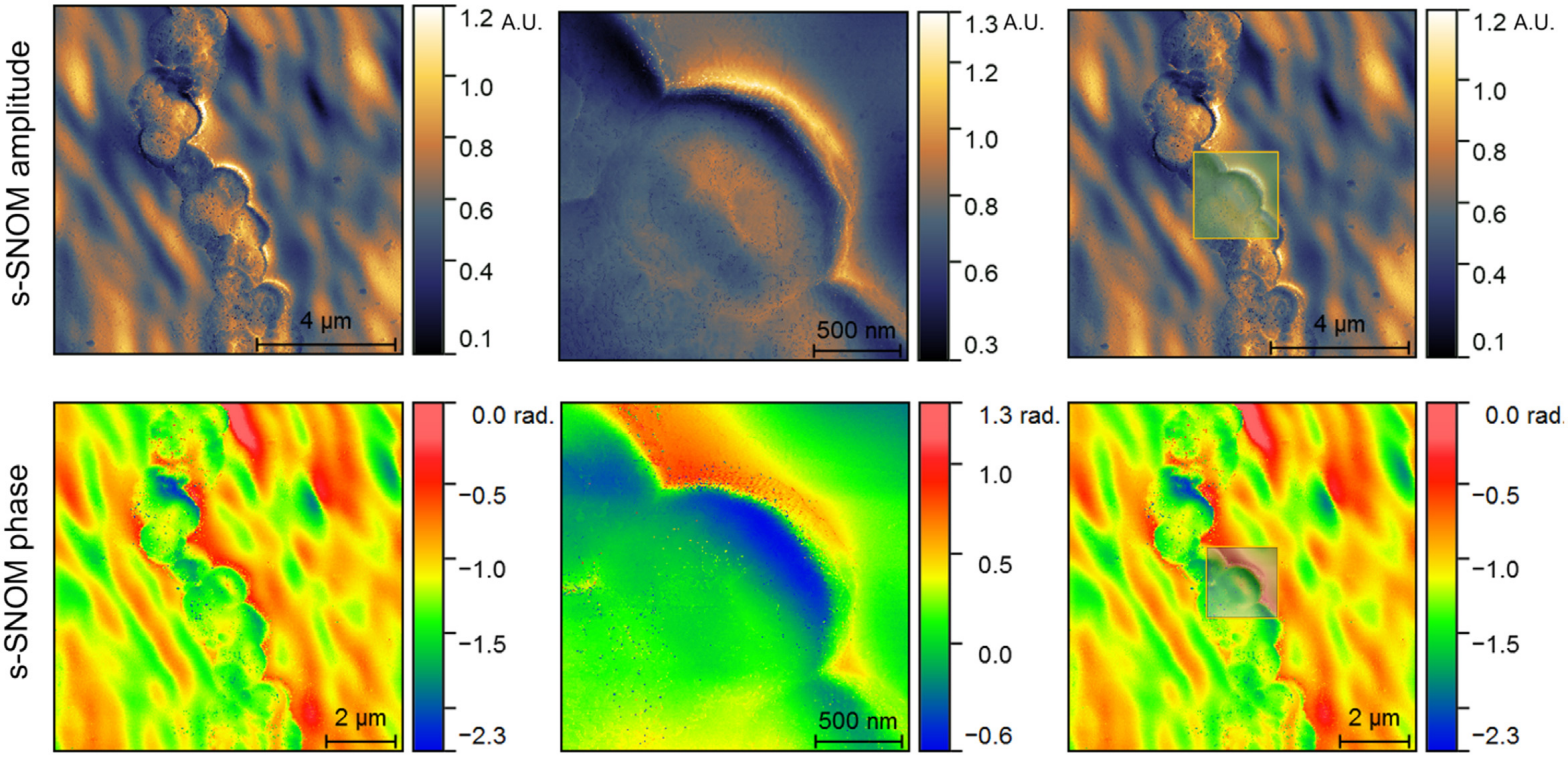
Example of cross-scale registration of s-SNOM amplitude and phase images collected on *A. baumannii* ATCC 17978 using an in-house–developed multi-scale mutual information–based registration approach. The result of the registration is visualized on the right as a transparent overlay.

Image acquisition of bacteria can be performed at different scales to allow for visualization and examination of details at different scales, such as imaging a group of bacteria at low magnification versus imaging a single bacterium at high magnification. These different imaging scales will of course cover different levels of detail, which can be merged to allow a more comprehensive view (and understanding) of the specimen. Furthermore, when high-magnification image acquisition of particular cells or features is performed in an unsupervised manner, identifying them in a group of many similar ones imaged at low magnification (which is many times necessary for context understanding, e.g., [98]) is time consuming and difficult. In these cases, the automated alignment of 2 or more images of the same scene collected at different magnifications, known as image registration [99], can be of great help. Within the context of SSNOMBACTER, registration can be performed between images of the same modality (e.g., AFM images acquired at different scales as in Fig. 6) or different modalities [100] (e.g., alignment of AFM topography image on an s-SNOM image). The result of such a registration will allow for fusion of information obtained from different imaging techniques and/or at various scales (e.g., [82]).

Furthermore, similar to the popular computer vision application of panorama creation [101, 102], image stitching applications are useful for visualizing microscopy FOVs larger than those available in an imaging system [103]. While SSNOMBACTER does not contain images depicting overlapping regions, which could be stitched to result in mosaics, it is nonetheless useful for developing and benchmarking such AFM- and s-SNOM–oriented algorithms. This can be done by synthetically generating image tiles with a degree of overlap, by controlled cropping of the available images.

## Conclusions

We introduce SSNOMBACTER, a collection of 4,400 images collected with s-SNOM and AFM modalities on 15 bacterial species, including harmless species regarded as model organisms as well as pathogens included in the ESKAPE group. By publishing this carefully crafted collection, our interest is 3-fold: (i) we wish to increase the awareness of relevant stakeholders in the life sciences field of this valuable imaging technique, s-SNOM; (ii) we wish to draw the attention of more groups active in the field of s-SNOM towards its huge potential for enabling novel high-impact studies and applications in microbiology; and (iii) we wish to offer to the computer vision community the means to interact with s-SNOM outputs, leading to the advent of novel s-SNOM–oriented methods for automated image analysis. With respect to the latter, we carried out a detailed discussion on relevant use-cases. In the future we plan to extend this collection of images to cover additional pathogens, imaged with multiple laser wavelenghts. We hope that our effort will inspire similar ones, originating from other groups, leading to wider availability of datasets collected on bacterial species with emerging imaging modalities that could enhance our current understanding of prokaryotic organisms.

## Availability of Supporting Data and Materials

All data supporting this work are openly available in the OSF Platform [49].

## Abbreviations

AFM: atomic force microscopy; ESKAPE: *Enterococcus faecium, Staphylococcus aureus,Klebsiella pneumoniae, Acinetobacter baumannii, Pseudomonas aeruginosa*, and *Enterobacter* species; FOV: field of view; .gsf: Gwyddion Simple Field file format; nano-FTIR: nanoscale Fourier transform infrared spectroscopy; RI: refractive index; s-SNOM: scattering-type scanning near-field optical microscopy; SRM: super-resolution microscopy; .tiff: tagged image file format.

## Competing Interests

The authors declare that they have no competing interests.

## Funding

The work of S.G.S., D.E.T., and R.H. was supported by UEFISCDI grant PN-III-P1–1.1-TE-2016–2147 (CORIMAG). The use of the Neaspec NeaSNOM Microscope was possible due to European Regional Development Fund through Competitiveness Operational Program 2014–2020, Priority axis 1, Project No. P_36_611, MySMIS code 107066,—INOVABIOMED. The work of D.U. was realized during his STSM visit to Rome Tre University supported by the COST Action CA15124 NEUBIAS. The work of M.L. was supported by the COST Action CA17121 COMULIS.

## Authors' Contributions

M.L., P.V., and G.C. designed this dataset in terms of bacterial species to be imaged. S.G.S., G.C., and G.A.S. designed the experiment in terms of imaging configurations. M.L. and A.M.H. prepared the samples, under the guidance of P.V., G.A.S., G.C., and S.G.S., whereas M.L. and D.E.T. collected the images. L.N. and R.H. verified the dataset for potential inconsistencies and helped in its organization. D.U. and S.G.S. identified use-cases of the dataset for developing s-SNOM–oriented computer vision applications. All authors wrote and reviewed the manuscript.

## Supplementary Material

giaa129_GIGA-D-20-00201_Original_Submission

giaa129_GIGA-D-20-00201_Revision_1

giaa129_GIGA-D-20-00201_Revision_2

giaa129_Response_to_Reviewer_Comments_Original_Submission

giaa129_Response_to_Reviewer_Comments_Revision_1

giaa129_Reviewer_1_Report_Original_Submission Chris Armit -- 7/16/2020 Reviewed

giaa129_Reviewer_2_Report_Original_Submission Joachim Heberle -- 7/27/2020 Reviewed

giaa129_Reviewer_2_Report_Revision_1 Joachim Heberle -- 10/24/2020 Reviewed
