# Supplementary material for: SSNOMBACTER: A collection of scattering-type scanning near-field optical microscopy and atomic force microscopy images of bacterial cells
Source: Gigascience. 2020 Nov 24;9(11):giaa129. doi: 10.1093/gigascience/giaa129 (PMC7684706; doi:10.1093/gigascience/giaa129)
Supplement: giaa129_GIGA-D-20-00201_Revision_1 [file giaa129_giga-d-20-00201_revision_1.pdf]

GigaScience

SSNOMBACTER: A collection of scattering-type Scanning Near-Field Optical Microscopy and Atomic Force Microscopy images of bacterial cells

--Manuscript Draft--

**Manuscript Number:**	GIGA-D-20-00201R1
**Full Title:**	SSNOMBACTER: A collection of scattering-type Scanning Near-Field Optical Microscopy and Atomic Force Microscopy images of bacterial cells
**Article Type:**	Data Note
**Funding Information:**	Unitatea Executiva pentru Finantarea Invatamantului Superior, a Cercetarii, Dezvoltarii si Inovarii (P1-1.1-TE-2016-2147) — Dr. Stefan G. Stanciu<br>European Cooperation in Science and Technology (CA17121) — Mr. Massimiliano Lucidi
**Abstract:**	Over the past years, a variety of imaging techniques operating at nanoscale resolution have been reported. These techniques have the potential to enrich our understanding of bacterial species relevant to human health, such as antibiotic-resistant pathogens. However, due to the novelty of these techniques, their use is still confined to addressing very particular applications, and their availability is severely limited due to associated costs and required expertise. Among these, scattering-type Scanning Near Field Optical Microscopy (s-SNOM) has been demonstrated as a powerful tool for exploring important optical properties at nanoscale resolution, depending only on the size of a sharp tip. Despite its huge potential to resolve aspects that cannot be tackled otherwise, the penetration of s-SNOM into the life sciences is still proceeding at slow pace, due to the reasons mentioned above. In this work we introduce SSNOMBACTER, a set of s-SNOM images collected on 15 bacterial species. These come accompanied by registered Atomic Force Microscopy (AFM) images, which are useful for placing nanoscale optical information in a relevant topographic context. We consider this dataset to be useful for amplifying the popularity of s-SNOM and for accelerating its penetration in life sciences. Furthermore, we consider this dataset to be useful for the development and benchmarking of image analysis tools dedicated to s-SNOM imaging, which are extremely scarce, despite the high need. In this latter context we discuss a series of image processing and analysis application where SSNOMBACTER could be of help.
**Corresponding Author:**	Stefan G. Stanciu<br>Universitatea Politehnica din Bucuresti<br>Bucharest, ROMANIA
**Corresponding Author Secondary Information:**	
**Corresponding Author's Institution:**	Universitatea Politehnica din Bucuresti
**Corresponding Author's Secondary Institution:**	
**First Author:**	Massimiliano Lucidi
**First Author Secondary Information:**	
**Order of Authors:**	Massimiliano Lucidi<br>Denis E. Tranca<br>Lorenzo Nichele<br>Devrim Unay<br>George A. Stanciu<br>Paolo Visca

Powered by Editorial Manager® and ProduXion Manager® from Aries Systems Corporation

	Alina Maria Holban
	Radu Hristu
	Gabriella Cincotti
	Stefan G. Stanciu
**Order of Authors Secondary Information:**	
**Response to Reviewers:**	The rebuttal letter has been formatted in rich text format to ensure clarity, and also contains graphical elements. Due to this, we have uploaded it as a Supplementary information file. It is available for download at the end of the submitted pdf. Furthermore, the manuscript parts that have been modified to address the comments/suggestions of the Reviewers are listed in red-color font in the manuscript file.
**Additional Information:**	
**Question**	**Response**
Are you submitting this manuscript to a special series or article collection?	No
**Experimental design and statistics**<br><br>Full details of the experimental design and statistical methods used should be given in the Methods section, as detailed in our Minimum Standards Reporting Checklist. Information essential to interpreting the data presented should be made available in the figure legends.<br><br>Have you included all the information requested in your manuscript?	Yes
**Resources**<br><br>A description of all resources used, including antibodies, cell lines, animals and software tools, with enough information to allow them to be uniquely identified, should be included in the Methods section. Authors are strongly encouraged to cite Research Resource Identifiers (RRIDs) for antibodies, model organisms and tools, where possible.<br><br>Have you included the information requested as detailed in our Minimum Standards Reporting Checklist?	Yes
**Availability of data and materials**	Yes

Powered by Editorial Manager® and ProduXion Manager® from Aries Systems Corporation

All datasets and code on which the conclusions of the paper rely must be either included in your submission or deposited in publicly available repositories (where available and ethically appropriate), referencing such data using a unique identifier in the references and in the "Availability of Data and Materials" section of your manuscript.<br><br>Have you have met the above requirement as detailed in our Minimum Standards Reporting Checklist?	

Powered by Editorial Manager® and ProduXion Manager® from Aries Systems Corporation

Manuscript

Click here to access/download;Manuscript;SSNOMBACTER_revision_tosubm

SSNOMBACTER: A collection of scattering-type Scanning Near-Field Optical Microscopy and Atomic Force Microscopy images of bacterial cells

M. Lucidi, D.E. Tranca, L. Nichele, D. Unay, G.A. Stanciu, P. Visca, A.M. Holban, R. Hristu, G. Cincotti*, S.G. Stanciu*

*Correspondence:

gabriella.cincotti@uniroma3.it

stefan.stanciu@cmmip-upb.org

Abstract:

Over the past years, a variety of imaging techniques operating at nanoscale resolution have been reported. These techniques have the potential to enrich our understanding of bacterial species relevant to human health, such as antibiotic-resistant pathogens. However, due to the novelty of these techniques, their use is still confined to addressing very particular applications, and their availability is severely limited due to associated costs and required expertise. Among these, scattering-type Scanning Near Field Optical Microscopy (s-SNOM) has been demonstrated as a powerful tool for exploring important optical properties at nanoscale resolution, depending only on the size of a sharp tip. Despite its huge potential to resolve aspects that cannot be tackled otherwise, the penetration of s-SNOM into the life sciences is still proceeding at slow pace, due to the reasons mentioned above. In this work we introduce SSNOMBACTER, a set of s-SNOM images collected on 15 bacterial species. These come accompanied by registered Atomic Force Microscopy (AFM) images, which are useful for placing nanoscale optical information in a relevant topographic context. We consider this dataset to be useful for amplifying the popularity of s-SNOM and for accelerating its penetration in life sciences. Furthermore, we consider this dataset to be useful for the development and benchmarking of image analysis tools dedicated to s-SNOM imaging, which are extremely scarce, despite the high need. In this latter context we discuss a series of image processing and analysis application where SSNOMBACTER could be of help.

Keywords: scattering-type scanning near-field optical microscopy, atomic force microscopy, bacterial pathogens, dataset, bioimaging.

1. Context

Bacterial pathogens surround us, being found not only in infected patients, but also in soil, water, wild and domestic animals, or food. While diseases caused by some pathogenic bacteria can be prevented by immunization or relieved by antibiotic therapy, others still represent a major public health problem accounting for tens of millions of deaths annually across the globe. Furthermore, some pathogenic species are considered as possible warfare agents, and carry military relevance [1]. Among the most dangerous pathogenic bacterial species in developed countries, are those comprised in the ESKAPE group (Enterococcus faecium, Staphylococcus aureus, Klebsiella pneumoniae, Acinetobacter baumannii, Pseudomonas aeruginosa, and Enterobacter species)[2]. These are among the most common bacterial pathogens in nosocomial infections, causing extensive morbidity and mortality, especially in critically ill and immunocompromised patients[3]. All these species are characterized by a high level of resistance to a variety of antibiotics [4], which recently prompted the World Health Organization to list ESKAPE pathogens among one of the greatest threats to human health, and to boost research on new effective drugs for treatment of antibiotic-resistant infections[5].

A complete and detailed characterization of different bacterial pathogens plays a fundamental role in many biomedical studies, related to bacterial infection diagnosis and treatment. The determination of morphology and other biophysical parameters could provide additional information about both cellular structures and biochemical properties of bacteria. These parameters allow an accurate characterization, which could be employed to discriminate pathogenic from harmless commensal bacteria. However, the major part of bacterial structures cannot be investigated in detail by using conventional microscopy techniques due to resolution limitations. For example, the lateral resolution that can be achieved by using such conventional microscopy techniques based on laser excitation (*e.g.* Confocal Laser Scanning Microscopy) is limited by the light diffraction phenomena to half the wavelength of the excitation light, which translates to a ~200nm resolution barrier. As a result, an exact understanding of fundamental structures and processes of bacteria at subcellular levels is yet to be achieved, higher resolution being necessary for elucidating aspects that are still not well comprehended[6, 7]. Stimulated Emission Depletion Microscopy (STED)[8], Fluorescence Photoactivation Localization Microscopy (PALM)[9] or Stochastic Optical Reconstruction Microscopy (STORM)[10] succeed in overcoming

the resolution limits imposed by diffraction, offering typical resolutions in the range of 30-100nm. However, such nanoscopy techniques based on super-resolved fluorescence face a series of important limitations due to the lack of chemical sensitivity and dependence on (very specific) fluorescent probes. In the case of biological samples, the advantages of fluorescence super-resolution microscopy (SRM) techniques come accompanied by a series of drawbacks related to the fact that exogenous and genetically engineered contrast agents can influence the phenotype (morphology, metabolism, motility, etc.) of the cells that are imaged, and can also lead to cytotoxicity and phototoxicity. Furthermore, recent studies suggest that unpredictable anomalous processes related to the SRM fluorophore distribution in biological samples exist[11]. Such limitations and concerns keep scientists motivated to innovate alternative ways of overcoming the diffraction barrier in the form of optical imaging techniques that do not require contrast agents (label-free).

Among the label-free optical nanoscopy techniques that have emerged over the past years, two prominent families can be easily distinguished: (a) near-field techniques based on the interaction of light and a sharp tip scanned across the sample surface, such as scattering-type Scanning Near-Field Optical microscopy (s-SNOM)[12], tip-enhanced Fluorescence (TEF)[13], tip-enhanced photoluminescence (TEPL)[14], tip-enhanced Raman Spectroscopy (TERS)[15], Photoinduced Force Microscopy (pi-FM)[16], or Photothermal Atomic Force Microscopy (photothermal-AFM)[17], and b) far-field techniques based on pump and probe strategies where two or more incident beams compete [18, 19] [20] [21]. All these label-free techniques hold great potential for advancing beyond the state-of-the-art the current understanding of the structural, chemical and optical features of biological samples (and also of advanced (bio)materials). However, due to their novelty, their use is still confined to addressing very specific applications, and their availability is severely limited due to associated costs and required expertise. Access to datasets collected with these techniques is also widely limited due to the same reasons, which translates to huge delays in transferring them to important applications that lie outside the scientific interest of the reduced number of scientific groups developing and using them. Furthermore, modern methods for automated image analysis that have taken the fields of bioimaging (and microscopy in general) by storm over the past few years[22-24], have had insignificant intersections with these emerging label-free modalities, due to the same reasons expressed above. With this effort, we aim to alleviate the situation by establishing a new trend for sharing relevant data sets collected with such

modalities, and other emerging or novel ones. In our view, this would be of great help for enlarging and overcoming the aforementioned bottlenecks.

In the context discussed under the previous paragraph, we focus our attention on s-SNOM, a generally applicable label-free method for surface characterizations at nanoscale resolution[12], whose working principles rely on a sharp tip that is scanned across the sample while being excited with a focused laser beam. The tip converts the incident radiation into a highly localized and enhanced near-field at the tip apex, which modifies both the amplitude and the phase of the scattered light. This process depends on the local dielectric properties of the sample [25], given the mutual perturbations occurring between the polarizabilities of the sample and the probe. Interferometric detection of the backscattered light yields thus nanoscale-resolved amplitude and phase images, which can reveal various important properties of nanostructured materials[12, 26]. Measuring the amplitude and the phase changes separately is of interest given that these two different signals contain complementary information about the sample. The amplitude shows the magnitude of the electric field enhancement at the tip apex, which can be quantified by the scattering efficiency, e.g. the number of photons reaching the detector. The phase of near-field signals are related to the complex optical constants using quasi-electrostatic theory[27] and importantly, in the landmark work of Stiegler et al.[28] it was shown that the near-field phase spectra of small particles correlate well with their far-field absorption spectra. Noteworthy, the scattered field is comprised of a series of terms [29], namely the incident field (i) scattered by the tip, (ii) scattered by the sample, (iii) scattered by the sample and then by the tip, (iv) scattered by the tip and then by the sample, and (v) scattered by the tip, then the sample and finally the tip again. The sample properties dictate the weight of each of these terms in the recorded signals, but for an s-SNOM configuration based on detection at higher harmonics of the tip's oscillation frequency, the incident field scattered by the sample should be discarded[29]. From the s-SNOM amplitude and phase images one can typically extract material contrasts instead of absolute values, but in the case of samples where a reference material is available next to unknown ones, absolute values of optical parameters (e.g. refractive index, reflectance, etc.) are also available[26].

The complex but reliable contrast mechanisms of s-SNOM enabled so far a wide range of discoveries in the condensed phase materials, and two-dimensional materials[30-39]. With respect to imaging biological species, a growing number of experiments demonstrate s-SNOM's usefulness to resolve various properties of biological samples, unavailable with other

techniques[40-43]. For example, in the recent work of L. Mesarosz et al[44], s-SNOM was used to assess the local IR absorption of single bacterial cells. In particular, illumination at 1660 cm^{-1} (6024 nm) was employed to visualize the protein content (amide I band) and distribution in a number of representative cells. In the work of Berweger et al.[45], the authors employed nano-FTIR spectroscopy (available with an s-SNOM system equipped with a broadband laser) to identify the distribution and density of the membrane protein bacteriorhodopsin (bR) in dried purple membrane patches purified from H. Salinarum. The authors demonstrated s-SNOM images at 20nm spatial resolution, with the s-SNOM phase images depicting contrast from the amide I vibrational mode of bR available under illumination with 1667 cm^{-1}. In a more recent experiment addressing s-SNOM imaging of the same purple membrane patches of H. Salinarum based on amide I band contrast, Pfitzner and Heberle[46] introduced a hardware configuration that allows imaging in liquid environments. This represents an especially important feature given that investigating biological samples in their native environment allows assessing various dynamic processes including structural changes and others. While in a different experiment the authors used s-SNOM to investigate the same amide bands of individual tobacco mosaic viruses and ferritin complexes, insulin aggregates and purple membranes[47], also a notable effort is that of Paulite et al[48], where s-SNOM was employed to assess the composition variations and secondary structure of individual amyloid fibrils, which result from the nucleation-dependent polymerization of proteins. In a different work showcasing the power of s-SNOM wrt. understanding important features of biochemical components, Kästner et al[49] showed that SNOM based nano-FTIR spectroscopy can identify and chemically detect domain formation in mixed phospholipid and surfactin monolayers at nanoscale. While these briefly surveyed studies base their conclusions on contrast observable in s-SNOM specific amplitude and phase images/spectra, we find important to mention that s-SNOM amplitude and phase images can be processed to result in maps of the real and imaginary parts of the refractive index (RI). In the previous work of Tranca et al[50], a proof-of-concept experiment focused on nanoscale RI mapping of erythrocytes showed that such properties (that are much more easy to interpret compared to raw s-SNOM data), are easily available with s-SNOM, supposing a ground-truth reference (a region of known RI) is available on the sample[50]. The utility of s-SNOM imaging for quantitative imaging of the dielectric function and connected optical parameters has been also showcased in a recent experiment in connection to various types of nanomaterials[26].

To facilitate the popularity of s-SNOM and promote new applications in life-sciences, we introduce here SSNOMBACTER[51], a collection of s-SNOM images assembled by imaging 15 bacterial species, including those in the ESKAPE group. These s-SNOM images come accompanied by registered Atomic Force Microscopy (AFM) data, intrinsically available in a s-SNOM imaging session, due to the underlying data acquisition principles. The s-SNOM - AFM image pairs are useful for placing nanoscale optical information in a relevant topographic context; the latter's importance for understanding the structure of bacteria is nicely presented in the recent landmark work of Pasquina-Lemonche et al. [52]. The potential uses of the dataset presented here include the topographical, biophysical and morphological analysis at nanoscale level of different bacterial species. The dataset includes the most representative reference strains of ESKAPE and cystic fibrosis-associated pathogens, including also *Streptococcus pyogenes*, an important human pathogen that causes a wide variety of acute morbidities (soft tissue infections and pharyngitis), severe life-threatening infections (i.e., streptococcal toxic shock syndrome) and devastating postinfectious sequelae such as rheumatic fever and glomerulonephritis[53]. Noteworthy, the proposed dataset has been assembled by imaging both Gram-positive and Gram-negative bacteria. With respect to the latter, we include in our dataset s-SNOM images collected on *Escherichia coli* strain, which still represents the most prominent model among Gram-negative bacteria. Given the diversity of information included in the SSNOMBACTER dataset (e.g., optical phase and amplitude, topography, morphology, etc.), we believe that it can potentially be useful to devise novel bacterial identification strategies that rely on combined s-SNOM/AFM datasets. For this purpose, additional Gram-positive species were incorporated in SSNOMBACTER, namely the Gram-positive model organism *Bacillus subtilis* and the commensal/opportunistic pathogen *Staphylococcus epidermidis*. In our view, all tested species could represent a relevant starting point to develop new s-SNOM/AFM image analysis workflows aimed at distinguishing commensal from pathogenic bacteria.

In the following, we describe how the dataset is structured, provide details on how the s-SNOM/AFM imaging was performed, and reflect on a series of computer vision applications where SSNOMBACTER would be useful to support and inspire the development of new s-SNOM oriented image analysis tools.

2. Methods

2.1. Bacterial sample preparation

The bacterial strains employed in this work are listed in Table 1. All the bacterial species were routinely grown on nutrient agar plates, except for *S. pyogenes* that was grown on blood agar plates. Three colonies of each bacterial strains were inoculated in Tryptic Soy Broth or in Todd-Hewitt broth for *S. pyogenes*, and incubated at 37°C for 24 hours under vigorous shaking (300 rpm in an orbital shaker). After the incubation, the bacterial cultures were centrifuged at 3,000 g x 5 min, washed twice and diluted in sterile distilled water to reach a final absorption at 600 nm (OD_{600}) =1. Twenty µl of each bacterial suspension at OD_{600}=1 were spotted on glass coverslip (Zeiss, Germany), with an refractive index of 1.5077 (@1550 nm) and air-dried under the laminar flow hood for 20 minutes at room temperature. After the desiccation, the samples were imaged with AFM/s-SNOM.

At least three different 10x10 µm field-of-views (FOV) were acquired including both glass substrate regions (employed as reference, required for potential quantitative s-SNOM image analyses[26, 50, 54]) and bacterial cells. In addition, at least one FOV was imaged at higher magnification (i.e., by scanning a region of lower dimension, namely of 2x2 µm or 4x4 µm). The considered FOV dimensions were selected depending on the dimensions of the selected species; in particular, the minimum FOV has been selected to include a single cell of the species under examination. The image dataset available for each of the considered bacterial species is summarized in Table 1.

Table 1. Bacterial strains and FOV configurations addressed in the proposed SSNOMBACTER dataset

**Bacterial strain**	**Gram**	**Bacterial strain reference**	**Number of imaged regions x FOV dimensions**
Achromobacter xylosoxidans ATCC 27061 (DSMZ2402) ^{T}	-	Yabuuchi and Oyama, 1971[55]	3 x (10 µm x 10 µm);<br>1 x (2 µm x 2 µm)

Acinetobacter baumannii ATCC 17978	-	Sahm et al., 1989[56]	4 x (10 µm x 10 µm); 1 x (2 µm x 2 µm)
Acinetobacter baumannii ATCC 19606[T]	-	American Type Culture Collection (Bouvet and Grimont, 1986)[57]	3 x (10 µm x 10 µm); 2 x (2 µm x 2 µm)
Bacillus subtilis subsp. *spizizenii* DSMZ 347	+	American Type Culture Collection	3 x (10 µm x 10 µm); 1 x (4 µm x 4 µm)
Burkholderia cenocepacia ATCC BAA-245 (LMG 16656) [T]	-	Govan et al., 1993[58]	3 x (10 µm x 10 µm); 1 x (2 µm x 2 µm)
Enterobacter aerogenes ATCC 13048 (DSMZ30053) [T]	-	Bascomb et al., 1971[59]	3 x (10 µm x 10 µm); 1 x (2 µm x 2 µm)
Enterobacter cloacae ATCC 13047 (DSMZ30054) [T]	-	Hormaeche and Edwards, 1960[60]	3 x (10 µm x 10 µm); 1 x (2 µm x 2 µm)
Enterococcus faecalis ATCC 29212	+	American Type Culture Collection	3 x (10 µm x 10 µm); 3 x (2 µm x 2 µm)
Enterococcus faecalis ATCC 700802 (V583)	+	Sahm et al., 1989[56]	3 x (10 µm x 10 µm); 1 x (2 µm x 2 µm)
Enterococcus faecium ATCC 19434 (DSMZ20477) [T]	+	Schleifer and Kilpper-Balz, 1984[61]	3 x (10 µm x 10 µm); 1 x (2 µm x 2 µm)

Escherichia coli MG1655 (ATCC700926) [T]	-	American Type Culture Collection	4 x (10 µm x 10 µm); 1 x (2 µm x 2 µm)
Klebsiella pneumoniae ATCC 27736	-	American Type Culture Collection	3 x (10 µm x 10 µm); 1 x (2 µm x 2 µm)
Pseudomonas aeruginosa PAO1 (ATCC 15692) [T]	-	American Type Culture Collection	3 x (10 µm x 10 µm); 2 x (2 µm x 2 µm)
Staphylococcus aureus ATCC 25923	+	American Type Culture Collection	4 x (10 µm x 10 µm); 1 x (2 µm x 2 µm)
Staphylococcus aureus ATCC 43300	+	American Type Culture Collection	3 x (10 µm x 10 µm); 1 x (2 µm x 2 µm)
Staphylococcus epidermidis SP1	+	Spallanzani hospital, clinical isolate	4 x (10 µm x 10 µm); 1 x (2 µm x 2 µm)
Stenotrophomonas maltophilia ATCC 13637 (DSMZ50170) [T]	-	Palleroni and, Bradbury, 1980[62]	4 x (10 µm x 10 µm); 1 x (3 µm x 3 µm)
Streptococcus pyogenes ATCC 19615	+	American Type Culture Collection	4 x (10 µm x 10 µm); 1 x (2 µm x 2 µm)

2.2 s-SNOM/AFM data acquisition

For acquiring the images available in the SSNOMBACTER dataset we used a NeaSNOM Microscope (Neaspec, Munich, Germany) equipped with a laser source of fixed wavelength, 1550nm. Importantly, s-SNOM configurations are available also with visible, IR and THz laser excitation sources[63-69], and also with broadband lasers that allow spectroscopic assays[47, 70].

While the wavelength that we have used (1550nm = 6451.61 cm^{-1}) has not been specifically chosen to match a particular optical property of the bacteria (e.g. the absorption properties of bacteria components lies elsewhere[71]), it provides nonetheless s-SNOM amplitude and phase images that correspond to the dielectric properties of the investigated sample, as discussed earlier. Furthermore, it is important to mention that despite the bacterial samples having been desiccated before imaging with AFM/s-SNOM, a small amount of water may have been however retained by these in response to the osmotic shock induced by dehydration[72]. Thus, some features in the s-SNOM phase images may be linked to the absorption of water molecules near/on the bacterial membrane. One additional important aspect to discuss is the non-invasive effect of the used wavelength. In a previous work[73], the effects of lasers with wavelengths ranging from 500 nm to 1550 nm were investigated on different bacterial species (i.e., the Gram-negative Escherichia coli and the three Gram-positive microorganisms Bacillus subtilis, Bacillus cereus and Micrococcus luteus), two of which are included in the database presented in our work (i.e., E. coli and B. subtilis). The 1550 nm laser line showed the lowest laser-induced cell lysis, preserving the intracellular RNA and minimizing the effects on intracellular enzymatic activity. Thus, the features available in the s-SNOM phase and amplitude images correspond to the intrinsic properties of desiccated bacteria (except for potential water contamination), and not to potential compounds that may result from phototoxicity.

In s-SNOM the available resolution is dictated by the size and geometry of the tip, and in the case of this experiment a Mikromasch Hq:NSC19/Cr-Au gold coated probe with <35nm tip radius was used. Its resonance frequency and force constant are 65 kHz, and 0.5 N/m, respectively.

3. Dataset structure

SSNOMBACTER is a dataset comprised of 4,400 images collected with AFM and s-SNOM in various workmodes, each of these made available in both tagged image file format (.tiff) and Gwyddion Simple Field file format (.gsf). The .tiff files can be opened with any image viewer/processing software, e.g. the freeware image viewer IrfanView or Image J, while the .gsf files represent the default file format of the NeaSNOM system that was used in this experiment for AFM/s-SNOM imaging. .gsf files can be accessed with the open-source Gwyddion software[74]. The collection of .tiff and .gsf files is divided in 15 folders, one for each of the bacterial species reported in Table 1. Some of the folders are further structured in sub-folders, depending on the

bacterial strains, which exist for some of the considered species. Each bacterial strain (or bacterial species) folder, harbors a number of subfolders that are numerically entitled. For ach sample at least three 10x10 µm FOVs were imaged, for this FOV dimension the names of these subfolders are equivalent to the number of the imaged sample region. For lower dimension FOVs (e.g. 2x2 µm or 4x4 µm), the FOV dimension (and FOV number) are explicitly presented in the subfolder name. The number of imaged FOVs for each specimen is presented in Table 1. For each FOV we provide data collected in complementary s-SNOM and AFM imaging modes, listed in Table 2.

The *.gsf and the corresponding *.tiff are entitled according to the following nomenclature:

[bacterial strain]_[FOV_number] [imaging_mode_abbreviation].

For instance, the filename "*Achromobacter xylosoxidans* ATCC 27061_1 M2A.gsf" indicates that the respective *.gsf file corresponds to the second harmonic of the topography error image, collected on the 1st FOV for *A. xylosoxidans* strain ATCC 27061. In each FOV folder we provide a *.txt file that presents all the acquisition parameters used for the respective measurement (i.e., pixel area, scan area, etc.). In Fig. 1, we provide a sample image subset consisting in AFM topography and phase, and s-SNOM amplitude and phase images collected on *Staphylococcus aureus* ATCC 25923.

Table 2. List of abbreviation of the AFM/s-SNOM imaging modes

**Abbreviation** [a]	**Corresponding description**
M0A	AFM Topography Error
M1A	AFM Topography Error; first harmonic
M2A	AFM Topography Error; second harmonic
M3A	AFM Topography Error; third harmonic
M4A	AFM Topography Error; fourth harmonic
M5A	AFM Topography Error; fifth harmonic
M1P	AFM Topography Phase; first harmonic
M2P	AFM Topography Phase; second harmonic
M3P	AFM Topography Phase; third harmonic
M4P	AFM Topography Phase; fourth harmonic
M5P	AFM Topography Phase; fifth harmonic

O0A	s-SNOM amplitude
O1A	s-SNOM amplitude, first harmonic
O2A	s-SNOM amplitude, second harmonic
O3A	s-SNOM amplitude, third harmonic
O4A	s-SNOM amplitude, fourth harmonic
O5A	s-SNOM amplitude, fifth harmonic
O0P	s-SNOM phase
O1P	s-SNOM phase, first harmonic
O2P	s-SNOM phase, second harmonic
O3P	s-SNOM phase, third harmonic
O4P	s-SNOM phase, fourth harmonic
O5P	s-SNOM phase, fifth harmonic
Z	AFM Topography

[a] The letter R reported in the dataset files indicates that the image was collected in the reverse scanning direction (e.g., RZ - topography collected in reverse scanning direction).

Each file of SSNOMBACTER[51] can be downloaded individually, or the whole set can be downloaded as a ZIP archive.

4. Re-use: utility of SSNOMBACTER for the development of s-SNOM oriented computer vision applications

The SSNOMBACTER dataset consists of 4,400 images collected with s-SNOM and AFM modalities on 15 bacterial species. The dimension of this dataset can be further expanded either by processing the available images to extract other types of data representations (e.g. by assembling 3D representations from the available AFM topographic information or by calculating dielectric function maps from the amplitude and phase s-SNOM images[26]). A different way to expand our dataset can rely on data augmentation strategies that apply various transformations to an initial image in order to render new representations that simulate other potential acquisition conditions. Such data augmentation strategies have been demonstrated as being particularly useful in Deep Learning approaches[75]. Given the content, dimension, and variability available in our data set, we envision that it represents a useful resource to develop novel image processing and analysis

tools dedicated to AFM and s-SNOM imaging, and benchmark existing ones. While such tools have already been reported for AFM imaging, they are still largely unavailable for the more recent s-SNOM modality, whose spread and number of applications have escalated over the past years[12]. The importance of reference datasets that enable objective comparisons between competing microscopy oriented image analysis/processing approaches is discussed in detail by R. Ulysse et al.[76]. In the following, we discuss potential use-cases of our dataset.

4.1 Image restoration and denoising

By employing digital restoration methods, an image whose quality is affected by noise, artefacts or improper acquisition conditions is processed to obtain a better estimate of the original object. The aforementioned causes are technique or equipment dependent, thus they greatly differ between imaging modalities that rely on optical and scanning probe principles. In the case of the latter, probe damage, mismatch between probe and sample geometry, scanner drift, vibrations, surface contamination and others impede an unbiased visualization of the imaged sample [77, 78]. Furthermore, these causes are further extended in s-SNOM by inconsistencies in the alignment of the s-SNOM excitation beam and the apex of the probe, which may occur while collecting an image[79]. Such inconsistencies translate to signal variations that raise problems with respect to manual and automated analysis of the recorded image. Moreover, interferences between near-field and background signals contained in the scattered field contributing to the image are also known to produce artifacts in s-SNOM[80].

The proposed data set can be used to develop novel image restoration methods oriented towards s-SNOM and AFM imaging and benchmark existing ones. We envision two potential scenarios for such efforts. In the first, the images available in our set can be restored, Fig. 2, and afterwards the quality of the corrected image can be evaluated by means of no-reference (blind) image quality algorithms[81]. In an alternative approach, the proposed images can be regarded as ground truth, and degraded instances can be synthetically generated. In this second case, as that ground-truth exists, the results of image restoration methods developed for addressing AFM and s-SNOM data can be evaluated by means of full-reference image quality assessment algorithms[82].

4.2 Quantitative imaging meets image fusion and correlative display

Quantitative imaging is especially important for achieving an in-depth understanding of both biological and materials samples. The availability of quantifiable features allows performing

objective analyses on the sample properties, and consequently drawing unbiased conclusions. While topographic information collected with AFM is intrinsically quantitative in nature, D.E. Tranca et al. demonstrated that s-SNOM images of the same FOV, but collected under different acquisition conditions (e.g. different modulation harmonics) or depicting complementary information (amplitude and phase), can be processed based on a methodology that relies on the oscillating point dipole model, in order to extract a nanoscale map of precise values of the dielectric function, and intrinsic optical properties (e.g. refractive index, absorption, reflectance, etc.)[50, 54], Fig. 3. In a follow-up study[26], the usefulness of such approaches was demonstrated in the case of different types of distinct nanomaterials. The availability of AFM and s-SNOM images enables the development and benchmarking of methods aimed at fusing and integrating these complementary information categories, which is useful for their joint visualization and analysis. The importance of software tools that jointly process and display topographic and optical data has been thoroughly discussed in the past[83-86].

4.3 Image segmentation

Image segmentation is the process of partitioning an image into sets of pixels known as segments in order to allow for easier and meaningful representation[87]. In bioimage analysis applications, image segmentation is a crucial task, which precedes further analyses carried out at the single structure (e.g. cell) level[88, 89]. Specific to the dataset proposed here, segmentation of bacteria can be realized by using images corresponding to a single or to multiple modalities. In the latter scenario, complementary information originated from entirely distinct modalities (e.g. AFM and s-SNOM) or from distinct but related contrasts of the same modality (e.g. amplitude and phase images of s-SNOM) can be employed to design segmentation algorithms with improved segmentation accuracy (Fig. 4). The availability of images of bacteria collected with different techniques or different contrast principles of the same techniques is also useful to support the development and benchmarking of generic algorithms that aim to be workmode invariant[90, 91]. Furthermore, it can support the development of adversarial methods that transfer knowledge[92, 93] learnt from a widely available imaging modality (e.g., confocal or brightfield) to one or more imaging modalities with reduced availability (e.g., s-SNOM) for which sufficient labeled training data is not available. SSNOMBACTER can thus help expand past work that has been done on

segmenting bacteria, addressing important tasks such as classification [94], proliferation and lineage analyses[95] and others [89, 96].

4.4 Image feature extraction

Previous studies have shown that morphological features such as shape, cell size and size distribution, cell wall thickness, and many others can be useful in distinguishing between various bacterial species, or between different types of the same species[97]. Measuring such properties manually is possible, but very tedious and time consuming. Fortunately, computer vision algorithms can be of great help for automating such tasks, but obviously they need to be developed and benchmarked using relevant data sets. SSNOMBACTER is comprised of both AFM and s-SNOM images of 15 bacterial species, collected at different scales, and hence can consistently support such efforts. The provided image sets are helpful in developing methods that automatically identify and extract various descriptive features, whose importance for various tasks has already been demonstrated, or for designing new features that can bring added value to important problems such as diagnostics, screening, etc. Furthermore, the proposed data set can also support the development of methods that exploit such descriptive features to answer various biologically motivated image analysis questions, such as "can we distinguish one bacterial species from the others by applying machine learning on images?" or "can we discriminate viable bacteria from the dead using image features only?". Such methods for automated classification/identification of different bacterial types can obviously be of immense help for saving time and human resources[98]. Noteworthy, given that the s-SNOM and AFM images are by default registered, SSNOMBACTER supports as well the development of computer vision algorithms capable to generalize, and hence to address dual-mode or multi-mode imaging applications[99]. Fig. 5 displays an exemplary use-case on identification of image features extracted from bacteria on AFM and s-SNOM data, where information like intensity profiles or size- and shape-related characteristics (e.g. area, roundness) from bacteria regions can be obtained. As aforementioned, such features can subsequently be used in various computer vision tasks promoting speed and efficiency.

4.5 Image registration and stitching

Image acquisition of bacteria can be performed at different scales to allow for visualization and examination of details at different scales, such as imaging a group of bacteria at low magnification versus imaging a single bacterium at high magnification. These different imaging scales will of

course cover different levels of detail, which can be merged to allow a more comprehensive view (and understanding) of the specimen. Furthermore, when high-magnification image acquisition of particular cells or features is performed in an unsupervised manner, identifying them in a group of many similar ones imaged at low magnification (which is many times necessary for context understanding, e.g. [100]), is time consuming and difficult. In these cases, the automated alignment of two or more images of the same scene collected at different magnifications, known as image registration[101], can be of great help. Within the context of SSNOMBACTER, registration can be performed between images of the same modality (e.g. AFM images acquired at different scales as in Fig. 6 or different modalities[102] (e.g. alignment of AFM topography image on an s-SNOM image). Result of such a registration will allow for fusion of information obtained from different imaging techniques and/or at various scales, e.g. [84].

Furthermore, similar to the popular computer vision application of panorama creation[103, 104], image stitching applications are very useful for visualizing microscopy FOVs larger than those available in an imaging system[105]. While SSNOMBACTER does not contain images depicting overlapping regions, which could be stitched to result in mosaics, it is nonetheless useful for developing and benchmarking such AFM and s-SNOM oriented algorithms. This can be done by synthetically generating image tiles with a degree of overlap, by controlled cropping of the available images.

5. Conclusions

We introduce SSNOMBACTER, a collection of 4,400 images collected with s-SNOM and AFM modalities on 15 bacterial species, including harmless species regarded as model organisms as well as pathogens included in the ESKAPE group. By publishing this carefully-crafted collection, our interest is threefold: *i*) we wish to increase the awareness of relevant stakeholders in the life sciences field to this valuable imaging technique, s-SNOM; *ii*) we wish to draw the attention of more groups active in the field of s-SNOM towards its huge potential for enabling novel high-impact studies and applications in microbiology, and *iii*) we wish offer to the computer vision community the means to interact with s-SNOM outputs, leading to the advent of novel s-SNOM oriented methods for automated image analysis. With respect to the latter, we carried a detailed discussion on relevant use-cases. In the future we plan to extend this collection of images to cover additional pathogens. Hopefully, our effort will inspire similar ones, originating from other groups,

leading to wider availability of datasets collected on bacterial species with emerging imaging modalities that could enhance our current understanding of prokaryotic organism.

Funding:

The work of S.G. Stanciu, D.E. Tranca and R. Hristu was supported by UEFISCDI grant PN-III-P1-1.1-TE-2016-2147 (CORIMAG). The use of the Neaspec NeaSNOM Microscope was possible due to European Regional Development Fund through Competitiveness Operational Program 2014-2020, Priority axis 1, Project No. P_36_611, MySMIS code 107066, - INOVABIOMED. The work of M. Lucidi was supported by the COST Action CA17121 COMULIS.

Author contributions:

M. Lucidi, P. Visca, G. Cincotii have designed this dataset in terms of bacterial species to be imaged. S.G. Stanciu, G. Cincotti and G.A. Stanciu have designed the experiment in terms of imaging configurations. M. Lucidi, and A.M Holban have prepared the samples, under the guidance of P. Visca, G.A. Stanciu, G. Cincotti and S.G. Stanciu. M. Lucidi and D.E. Tranca have collected the images. L. Nichele and R. Hristu have verified the dataset for potential inconsistencies and helped in its organization. D. Unay and S.G. Stanciu have identified use-cases of the data set for developing s-SNOM oriented computer vision applications. All authors wrote and reviewed the manuscript.

References

1. Ivnitski D, Abdel-Hamid I, Atanasov P and Wilkins E. Biosensors for detection of pathogenic bacteria. Biosensors and Bioelectronics. 1999;14 7:599-624.
2. Boucher HW, Talbot GH, Bradley JS, Edwards JE, Gilbert D, Rice LB, et al. Bad bugs, no drugs: no ESKAPE! An update from the Infectious Diseases Society of America. Clinical infectious diseases. 2009;48 1:1-12.
3. Rice LB. Progress and challenges in implementing the research on ESKAPE pathogens. Infection Control & Hospital Epidemiology. 2010;31 S1:S7-S10.
4. Pendleton JN, Gorman SP and Gilmore BF. Clinical relevance of the ESKAPE pathogens. Expert review of anti-infective therapy. 2013;11 3:297-308.
5. Tacconelli E, Carrara E, Savoldi A, Harbarth S, Mendelson M, Monnet DL, et al. Discovery, research, and development of new antibiotics: the WHO priority list of antibiotic-resistant bacteria and tuberculosis. The Lancet Infectious Diseases. 2018;18 3:318-27.
6. Gahlmann A and Moerner W. Exploring bacterial cell biology with single-molecule tracking and super-resolution imaging. Nature Reviews Microbiology. 2014;12 1:9-22.
7. Cattoni D, Fiche J and Nöllmann M. Single-molecule super-resolution imaging in bacteria. Current opinion in microbiology. 2012;15 6:758-63.

8. Hell SW and Wichmann J. Breaking the Diffraction Resolution Limit by Stimulated-Emission - Stimulated-Emission-Depletion Fluorescence Microscopy. Opt Lett. 1994;19 11:780-2. doi:Doi 10.1364/Ol.19.000780.
9. Betzig E, Patterson GH, Sougrat R, Lindwasser OW, Olenych S, Bonifacino JS, et al. Imaging intracellular fluorescent proteins at nanometer resolution. Science. 2006;313 5793:1642-5. doi:DOI 10.1126/science.1127344.
10. Rust MJ, Bates M and Zhuang XW. Sub-diffraction-limit imaging by stochastic optical reconstruction microscopy (STORM). Nat Methods. 2006;3 10:793-5. doi:Doi 10.1038/Nmeth929.
11. Cosentino M, Canale C, Bianchini P and Diaspro A. AFM-STED correlative nanoscopy reveals a dark side in fluorescence microscopy imaging. Science advances. 2019;5 6:eaav8062.
12. Chen X, Hu D, Mescall R, You G, Basov D, Dai Q, et al. Modern Scattering- Type Scanning Near- Field Optical Microscopy for Advanced Material Research. Advanced Materials. 2019:1804774.
13. Gerton JM, Wade LA, Lessard GA, Ma Z and Quake SR. Tip-enhanced fluorescence microscopy at 10 nanometer resolution. Physical Review Letters. 2004;93 18:180801.
14. Yang B, Chen G, Ghafoor A, Zhang Y, Zhang Y, Zhang Y, et al. Sub-nanometre resolution in single-molecule photoluminescence imaging. Nature Photonics. 2020:1-7.
15. Stöckle RM, Suh YD, Deckert V and Zenobi R. Nanoscale chemical analysis by tip-enhanced Raman spectroscopy. Chemical Physics Letters. 2000;318 1-3:131-6.
16. Nowak D, Morrison W, Wickramasinghe HK, Jahng J, Potma E, Wan L, et al. Nanoscale chemical imaging by photoinduced force microscopy. Science advances. 2016;2 3:e1501571.
17. Lu F, Jin M and Belkin MA. Tip-enhanced infrared nanospectroscopy via molecular expansion force detection. Nature photonics. 2014;8 4:307-12.
18. Wang P, Slipchenko MN, Mitchell J, Yang C, Potma EO, Xu X, et al. Far-field imaging of non-fluorescent species with subdiffraction resolution. Nature photonics. 2013;7 6:449-53.
19. Zanini G, Korobchevskaya K, Deguchi T, Diaspro A and Bianchini P. Label-Free Optical Nanoscopy of Single Layer Graphene. ACS nano. 2019.
20. Tzang O, Pevzner A, Marvel RE, Haglund RF and Cheshnovsky O. Super-resolution in label-free photomodulated reflectivity. Nano letters. 2015;15 2:1362-7.
21. Gong L, Zheng W, Ma Y and Huang Z. Saturated Stimulated-Raman-Scattering Microscopy for Far-Field Superresolution Vibrational Imaging. Physical Review Applied. 2019;11 3:034041.
22. Meijering E, Carpenter AE, Peng H, Hamprecht FA and Olivo-Marin J-C. Imagining the future of bioimage analysis. Nature biotechnology. 2016;34 12:1250.
23. Pradhan P, Guo S, Ryabchykov O, Popp J and Bocklitz TW. Deep learning a boon for biophotonics? Journal of Biophotonics. 2020:e201960186.
24. Möckl L, Roy AR and Moerner W. Deep learning in single-molecule microscopy: fundamentals, caveats, and recent developments. Biomedical Optics Express. 2020;11 3:1633-61.
25. Keilmann F and Hillenbrand R. Near-field microscopy by elastic light scattering from a tip. PHILOSOPHICAL TRANSACTIONS-ROYAL SOCIETY OF LONDON SERIES A MATHEMATICAL PHYSICAL AND ENGINEERING SCIENCES. 2004:787-806.

26. Stanciu SG, Tranca DE, Pastorino L, Boi S, Song YM, Yoo YJ, et al. Characterization of Nanomaterials by Locally Determining their Complex Permittivity with Scattering-Type Scanning Near Field Optical Microscopy. ACS Applied Nano Materials. 2020.
27. Hillenbrand R and Keilmann F. Complex optical constants on a subwavelength scale. Physical Review Letters. 2000;85 14:3029.
28. Stiegler JM, Abate Y, Cvitkovic A, Romanyuk YE, Huber AJ, Leone SR, et al. Nanoscale infrared absorption spectroscopy of individual nanoparticles enabled by scattering-type near-field microscopy. Acs Nano. 2011;5 8:6494-9.
29. Deutsch B, Hillenbrand R and Novotny L. Near-field amplitude and phase recovery using phase-shifting interferometry. Optics express. 2008;16 2:494-501.
30. Qazilbash MM, Brehm M, Chae B-G, Ho P-C, Andreev GO, Kim B-J, et al. Mott transition in VO2 revealed by infrared spectroscopy and nano-imaging. Science. 2007;318 5857:1750-3.
31. Fei Z, Rodin A, Andreev G, Bao W, McLeod A, Wagner M, et al. Gate-tuning of graphene plasmons revealed by infrared nano-imaging. arXiv preprint arXiv:12024993. 2012.
32. Amenabar I, Poly S, Nuansing W, Hubrich EH, Govyadinov AA, Huth F, et al. Structural analysis and mapping of individual protein complexes by infrared nanospectroscopy. Nature communications. 2013;4.
33. Liu M, Wagner M, Abreu E, Kittiwatanakul S, McLeod A, Fei Z, et al. Anisotropic electronic state via spontaneous phase separation in strained vanadium dioxide films. Physical review letters. 2013;111 9:096602.
34. Westermeier C, Cernescu A, Amarie S, Liewald C, Keilmann F and Nickel B. Sub-micron phase coexistence in small-molecule organic thin films revealed by infrared nano-imaging. Nature communications. 2014;5:4101.
35. Yoxall E, Schnell M, Nikitin AY, Txoperena O, Woessner A, Lundeberg MB, et al. Direct observation of ultraslow hyperbolic polariton propagation with negative phase velocity. Nature Photonics. 2015;9 10:674.
36. Chen JN, Badioli M, Alonso-Gonzalez P, Thongrattanasiri S, Huth F, Osmond J, et al. Optical nano-imaging of gate-tunable graphene plasmons. Nature. 2012;487 7405:77-81. doi:10.1038/nature11254.
37. Fei Z, Rodin AS, Andreev GO, Bao W, McLeod AS, Wagner M, et al. Gate-tuning of graphene plasmons revealed by infrared nano-imaging. Nature. 2012;487 7405:82-5. doi:10.1038/nature11253.
38. Neuman T, Alonso- González P, Garcia- Etxarri A, Schnell M, Hillenbrand R and Aizpurua J. Mapping the near fields of plasmonic nanoantennas by scattering- type scanning near- field optical microscopy. Laser & Photonics Reviews. 2015;9 6:637-49.
39. Yao Z, Xu S, Hu D, Chen X, Dai Q and Liu M. Nanoimaging and Nanospectroscopy of Polaritons with Time Resolved s- SNOM. Advanced Optical Materials. 2019:1901042.
40. Khatib O, Wood JD, McLeod AS, Goldflam MD, Wagner M, Damhorst GL, et al. Graphene-based platform for infrared near-field nanospectroscopy of water and biological materials in an aqueous environment. ACS nano. 2015;9 8:7968-75.
41. Tranca D, Stanciu S, Hristu R, Witgen B and Stanciu GA. Nanoscale mapping of refractive index by using scattering-type Scanning Near-Field Optical Microscopy. Nanomedicine: Nanotechnology, Biology and Medicine. 2017.
42. Brehm M, Taubner T, Hillenbrand R and Keilmann F. Infrared spectroscopic mapping of single nanoparticles and viruses at nanoscale resolution. Nano Letters. 2006;6 7:1307-10.

43. Stanciu SG, Tranca DE, Hristu R and Stanciu GA. Correlative imaging of biological tissues with apertureless scanning near-field optical microscopy and confocal laser scanning microscopy. Biomedical optics express. 2017;8 12:5374-83.
44. Mészáros LS, Ceccaldi P, Lorenzi M, Redman HJ, Pfitzner E, Heberle J, et al. Spectroscopic investigations under whole-cell conditions provide new insight into the metal hydride chemistry of [FeFe]-hydrogenase. Chemical Science. 2020;11 18:4608-17.
45. Berweger S, Nguyen DM, Muller EA, Bechtel HA, Perkins TT and Raschke MB. Nano-chemical infrared imaging of membrane proteins in lipid bilayers. Journal of the American Chemical Society. 2013;135 49:18292-5.
46. Pfitzner E and Heberle J. Infrared Scattering-Type Scanning Near-Field Optical Microscopy of Biomembranes in Water. The Journal of Physical Chemistry Letters. 2020.
47. Amenabar I, Poly S, Nuansing W, Hubrich EH, Govyadinov AA, Huth F, et al. Structural analysis and mapping of individual protein complexes by infrared nanospectroscopy. Nature communications. 2013;4 1:1-9.
48. Paulite M, Fakhraai Z, Li IT, Gunari N, Tanur AE and Walker GC. Imaging secondary structure of individual amyloid fibrils of a β2-microglobulin fragment using near-field infrared spectroscopy. Journal of the American Chemical Society. 2011;133 19:7376-83.
49. Kästner B, Johnson CM, Hermann P, Kruskopf M, Pierz K, Hoehl A, et al. Infrared nanospectroscopy of phospholipid and surfactin monolayer domains. ACS omega. 2018;3 4:4141-7.
50. Tranca DE, Stanciu SG, Hristu R, Witgen BM and Stanciu GA. Nanoscale mapping of refractive index by using scattering-type Scanning Near-Field Optical Microscopy. Nanomedicine: Nanotechnology, Biology and Medicine. 2018;14 1:47-50.
51. Open Science Foundation, 2020. https://osf.io/5u6pg/?view_only=99c71e31a91940e0b374aee7fa0043ae.
52. Pasquina-Lemonche L, Burns J, Turner R, Kumar S, Tank R, Mullin N, et al. The architecture of the Gram-positive bacterial cell wall. Nature. 2020:1-4.
53. Henningham A, Barnett TC, Maamary PG and Walker MJ. Pathogenesis of group A streptococcal infections. Discovery medicine. 2012;13 72:329-42.
54. Tranca DE, Stanciu SG, Hristu R, Stoichita C, Tofail S and Stanciu GA. High-resolution quantitative determination of dielectric function by using scattering scanning near-field optical microscopy. Scientific reports. 2015;5:11876.
55. Yabuuchi E and Ohyama A. Achromobacter xylosoxidans n. sp. from human ear discharge. Japanese journal of microbiology. 1971;15 5:477-81.
56. Sahm DF, Kissinger J, Gilmore MS, Murray PR, Mulder R, Solliday J, et al. In vitro susceptibility studies of vancomycin-resistant Enterococcus faecalis. Antimicrobial agents and chemotherapy. 1989;33 9:1588-91.
57. Bouvet PJ and Grimont PA. Taxonomy of the genus Acinetobacter with the recognition of Acinetobacter baumannii sp. nov., Acinetobacter haemolyticus sp. nov., Acinetobacter johnsonii sp. nov., and Acinetobacter junii sp. nov. and emended descriptions of Acinetobacter calcoaceticus and Acinetobacter lwoffii. International Journal of Systematic and Evolutionary Microbiology. 1986;36 2:228-40.
58. Govan JR, Doherty C, Nelson J, Brown PH, Greening A, Maddison J, et al. Evidence for transmission of Pseudomonas cepacia by social contact in cystic fibrosis. The lancet. 1993;342 8862:15-9.

59. Bascomb S, Lapage S, Willcox W and Curtis M. Numerical classification of the tribe Klebsielleae. Microbiology. 1971;66 3:279-95.
60. Hormaeche E and Edwards P. Proposal for the Rejection of the Generic Name Cloaca Castellani and Chalmers, and Proposal of Enterobacter as a Generic Name with Designation of Type Species and of its Type Culture. With Request for an Opinion. International bulletin of bacteriological nomenclature and taxonomy. 1960;10 2:75-6.
61. Schleifer KH and Kilpper-Bälz R. Transfer of Streptococcus faecalis and Streptococcus faecium to the genus Enterococcus nom. rev. as Enterococcus faecalis comb. nov. and Enterococcus faecium comb. nov. International Journal of Systematic and Evolutionary Microbiology. 1984;34 1:31-4.
62. Palleroni NJ and Bradbury JF. Stenotrophomonas, a new bacterial genus for Xanthomonas maltophilia (Hugh 1980) Swings et al. 1983. International Journal of Systematic and Evolutionary Microbiology. 1993;43 3:606-9.
63. Taubner T, Hillenbrand R and Keilmann F. Performance of visible and mid- infrared scattering- type near- field optical microscopes. Journal of microscopy. 2003;210 3:311-4.
64. Huber AJ, Keilmann F, Wittborn J, Aizpurua J and Hillenbrand R. Terahertz near-field nanoscopy of mobile carriers in single semiconductor nanodevices. Nano letters. 2008;8 11:3766-70.
65. Von Ribbeck H-G, Brehm M, Van der Weide D, Winnerl S, Drachenko O, Helm M, et al. Spectroscopic THz near-field microscope. Optics express. 2008;16 5:3430-8.
66. Keilmann F and Amarie S. Mid-infrared frequency comb spanning an octave based on an Er fiber laser and difference-frequency generation. Journal of Infrared, Millimeter, and Terahertz Waves. 2012;33 5:479-84.
67. Jacob R, Winnerl S, Fehrenbacher M, Bhattacharyya J, Schneider H, Wenzel MT, et al. Intersublevel spectroscopy on single InAs-quantum dots by terahertz near-field microscopy. Nano letters. 2012;12 8:4336-40.
68. Bensmann S, Gaußmann F, Lewin M, Wüppen J, Nyga S, Janzen C, et al. Near-field imaging and spectroscopy of locally strained GaN using an IR broadband laser. Optics express. 2014;22 19:22369-81.
69. Keilmann F, Huber AJ and Hillenbrand R. Nanoscale conductivity contrast by scattering-type near-field optical microscopy in the visible, infrared and THz domains. Journal of Infrared, Millimeter, and Terahertz Waves. 2009;30 12:1255-68.
70. Huth F, Govyadinov A, Amarie S, Nuansing W, Keilmann F and Hillenbrand R. Nano-FTIR absorption spectroscopy of molecular fingerprints at 20 nm spatial resolution. Nano letters. 2012;12 8:3973-8.
71. Naumann D. Infrared spectroscopy in microbiology. Encyclopedia of Analytical Chemistry: Applications, Theory and Instrumentation. 2006.
72. Lebre PH, De Maayer P and Cowan DA. Xerotolerant bacteria: surviving through a dry spell. Nature Reviews Microbiology. 2017;15 5:285-96.
73. Dhawan MD, Wise F and Baeumner AJ. Development of a laser-induced cell lysis system. Analytical and bioanalytical chemistry. 2002;374 3:421-6.
74. Nečas D and Klapetek P. Gwyddion: an open-source software for SPM data analysis. Open Physics. 2012;10 1:181-8.
75. Shorten C and Khoshgoftaar TM. A survey on image data augmentation for deep learning. Journal of Big Data. 2019;6 1:60.

76. Rubens U, Mormont R, Paavolainen L, Bäcker V, Pavie B, Scholz LA, et al. BIAFLOWS: A collaborative framework to reproducibly deploy and benchmark bioimage analysis workflows. Patterns. 2020:100040.
77. Canale C, Torre B, Ricci D and Braga PC. Recognizing and avoiding artifacts in atomic force microscopy imaging. Atomic force microscopy in biomedical research. Springer; 2011. p. 31-43.
78. Schwarz U, Haefke H, Reimann P and Güntherodt HJ. Tip artefacts in scanning force microscopy. Journal of Microscopy. 1994;173 3:183-97.
79. Stanciu SG, Hristu R and Stanciu GA. Digital image inpainting and microscopy imaging. Microscopy research and technique. 2011;74 11:1049-57.
80. Schnell M, Carney PS and Hillenbrand R. Synthetic optical holography for rapid nanoimaging. Nature communications. 2014;5:3499.
81. Kamble V and Bhurchandi K. No-reference image quality assessment algorithms: A survey. Optik. 2015;126 11-12:1090-7.
82. Sheikh HR, Sabir MF and Bovik AC. A statistical evaluation of recent full reference image quality assessment algorithms. IEEE Transactions on image processing. 2006;15 11:3440-51.
83. Rajwa B, Mcnally HA, Varadharajan P, Sturgis J and Robinson JP. AFM/CLSM data visualization and comparison using an open- source toolkit. Microscopy research and technique. 2004;64 2:176-84.
84. Kondra S, Laishram J, Ban J, Migliorini E, Di Foggia V, Lazzarino M, et al. Integration of confocal and atomic force microscopy images. Journal of neuroscience methods. 2009;177 1:94-107.
85. Caplan J, Niethammer M, Taylor II RM and Czymmek KJ. The power of correlative microscopy: multi-modal, multi-scale, multi-dimensional. Current opinion in structural biology. 2011;21 5:686-93.
86. Walter A, Paul-Gilloteaux P, Plochberger B, Sefc L, Verkade P, Mannheim JG, et al. Correlated Multimodal Imaging in Life Sciences: Expanding the Biomedical Horizon. Frontiers in Physics. 2020;8:47.
87. Pal NR and Pal SK. A review on image segmentation techniques. Pattern recognition. 1993;26 9:1277-94.
88. Meijering E. Cell segmentation: 50 years down the road [life sciences]. IEEE Signal Processing Magazine. 2012;29 5:140-5.
89. Ducret A, Quardokus EM and Brun YV. MicrobeJ, a tool for high throughput bacterial cell detection and quantitative analysis. Nature microbiology. 2016;1 7:1-7.
90. Falk T, Mai D, Bensch R, Çiçek Ö, Abdulkadir A, Marrakchi Y, et al. U-Net: deep learning for cell counting, detection, and morphometry. Nature methods. 2019;16 1:67-70.
91. Yuan W, Wei J, Wang J, Ma Q and Tasdizen T. Unified generative adversarial networks for multimodal segmentation from unpaired 3D medical images. Medical Image Analysis. 2020:101731.
92. Yang Q, Zhang Y, Dai W and Pan SJ. Transfer learning. Cambridge University Press; 2020.
93. Weiss K, Khoshgoftaar TM and Wang D. A survey of transfer learning. Journal of Big data. 2016;3 1:9.

94. Song Y, He L, Zhou F, Chen S, Ni D, Lei B, et al. Segmentation, splitting, and classification of overlapping bacteria in microscope images for automatic bacterial vaginosis diagnosis. IEEE journal of biomedical and health informatics. 2016;21 4:1095-104.
95. Stylianidou S, Brennan C, Nissen SB, Kuwada NJ and Wiggins PA. SuperSegger: robust image segmentation, analysis and lineage tracking of bacterial cells. Molecular microbiology. 2016;102 4:690-700.
96. Kulwa F, Li C, Zhao X, Cai B, Xu N, Qi S, et al. A State-of-the-Art Survey for Microorganism Image Segmentation Methods and Future Potential. IEEE Access. 2019;7:100243-69.
97. Trattner S, Greenspan H, Tepper G and Abboud S. Automatic identification of bacterial types using statistical imaging methods. IEEE transactions on medical imaging. 2004;23 7:807-20.
98. Danuser G. Computer vision in cell biology. Cell. 2011;147 5:973-8.
99. Würflinger T, Stockhausen J, Meyer-Ebrecht D and Böcking A. Robust automatic coregistration, segmentation, and classification of cell nuclei in multimodal cytopathological microscopic images. Computerized Medical Imaging and Graphics. 2004;28 1-2:87-98.
100. Reinhard S, Aufmkolk S, Sauer M and Doose S. Registration and Visualization of Correlative Super-Resolution Microscopy Data. Biophysical journal. 2019;116 11:2073-8.
101. Zitova B and Flusser J. Image registration methods: a survey. Image and vision computing. 2003;21 11:977-1000.
102. Hu Z, Fan Z, Liu C, Wu Y and Wang C. Geometrical Patterns Based Cross-scale Image Registration for AFM and Optical Microscopy. In: *2019 IEEE International Conference on Manipulation, Manufacturing and Measurement on the Nanoscale (3M-NANO)* 2019, pp.276-80. IEEE.
103. Brown M and Lowe DG. Automatic panoramic image stitching using invariant features. International journal of computer vision. 2007;74 1:59-73.
104. Szeliski R. Image alignment and stitching: A tutorial. Foundations and Trends® in Computer Graphics and Vision. 2007;2 1:1-104.
105. Chalfoun J, Majurski M, Blattner T, Bhadriraju K, Keyrouz W, Bajcsy P, et al. Mist: accurate and scalable microscopy image stitching tool with stage modeling and error minimization. Scientific reports. 2017;7 1:4988.

Figures

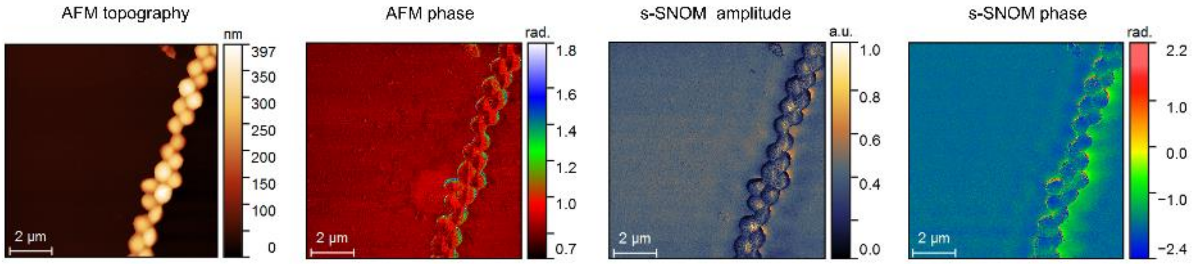

Fig 1: *Sample AFM and s-SNOM images collected on S. aureus ATCC 25923. The AFM phase image corresponds to the 1st harmonic of the tip's tapping frequency (M1P), the s-SNOM amplitude and phase images correspond to the 3rd harmonic of the tip's tapping frequency (O3A, O3P).*

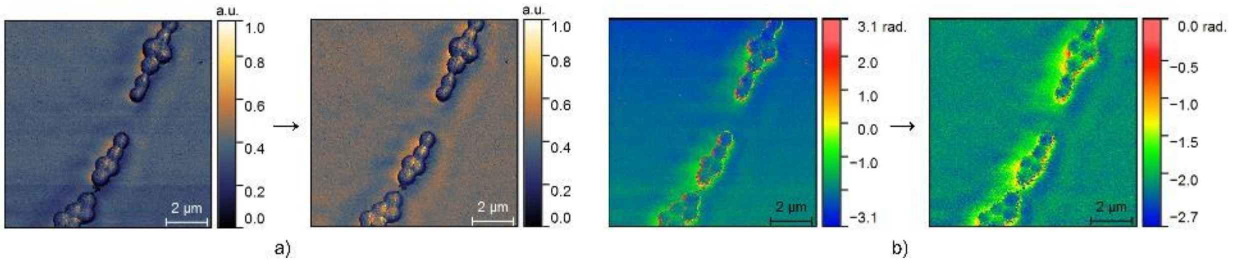

Fig 2: *Restoration of s-SNOM data by digital image processing. The raw s-SNOM amplitude (a) and phase image (b) collected on S. aureus ATCC 25923 have been processed in the Gwyddion software with three operations: "Align rows by median", "Correction of horizontal strokes", "Correct small grains marked by >90% threshold by interpolation". The resulted s-SNOM images (right of arrow) have homogeneous background, and the bacterial cells are displayed with better contrast.*

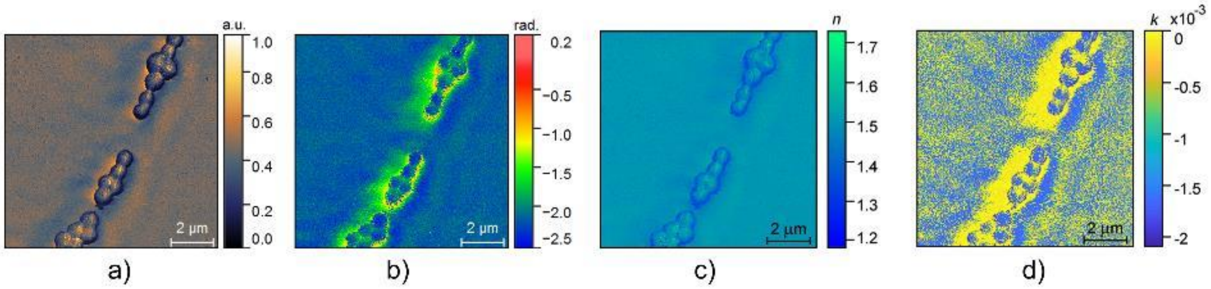

Fig 3: *Quantitative representation of the refractive index (imaginary part and real part) assembled using s-SNOM amplitude and phase images collected on S. aureus ATCC 25923 under different settings, using a previously reported methodology[50]. a) s-SNOM amplitude (O3A); b) s-SNOM phase (O3P); c) refractive index real part (n); d) refractive index imaginary part (k) (a.k.a extinction coefficient).*

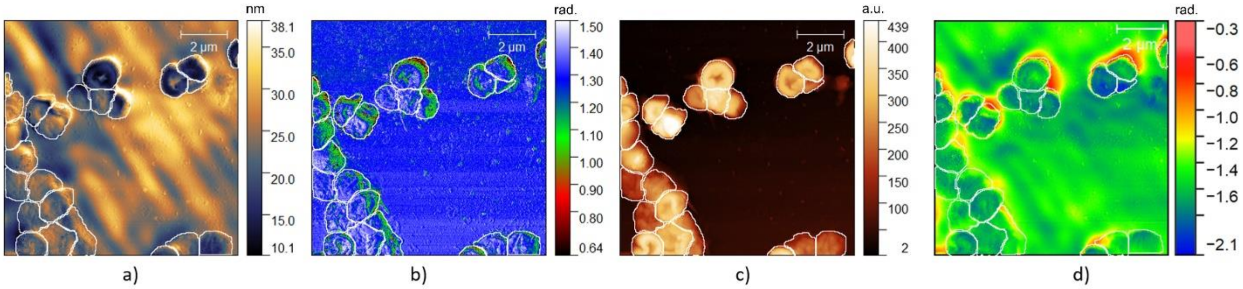

Fig. 4: *Multi-modality segmentation example of amplitude and phase images of s-SNOM and AFM collected on Acinetobacter baumannii ATCC 17978. The boundaries of the bacteria present in the field-of-view are manually delineated and visualized as overlays using the publicly available ImageJ/FIJI program. a) AFM amplitude; b) AFM phase; c) s-SNOM amplitude; d) s-SNOM phase.*

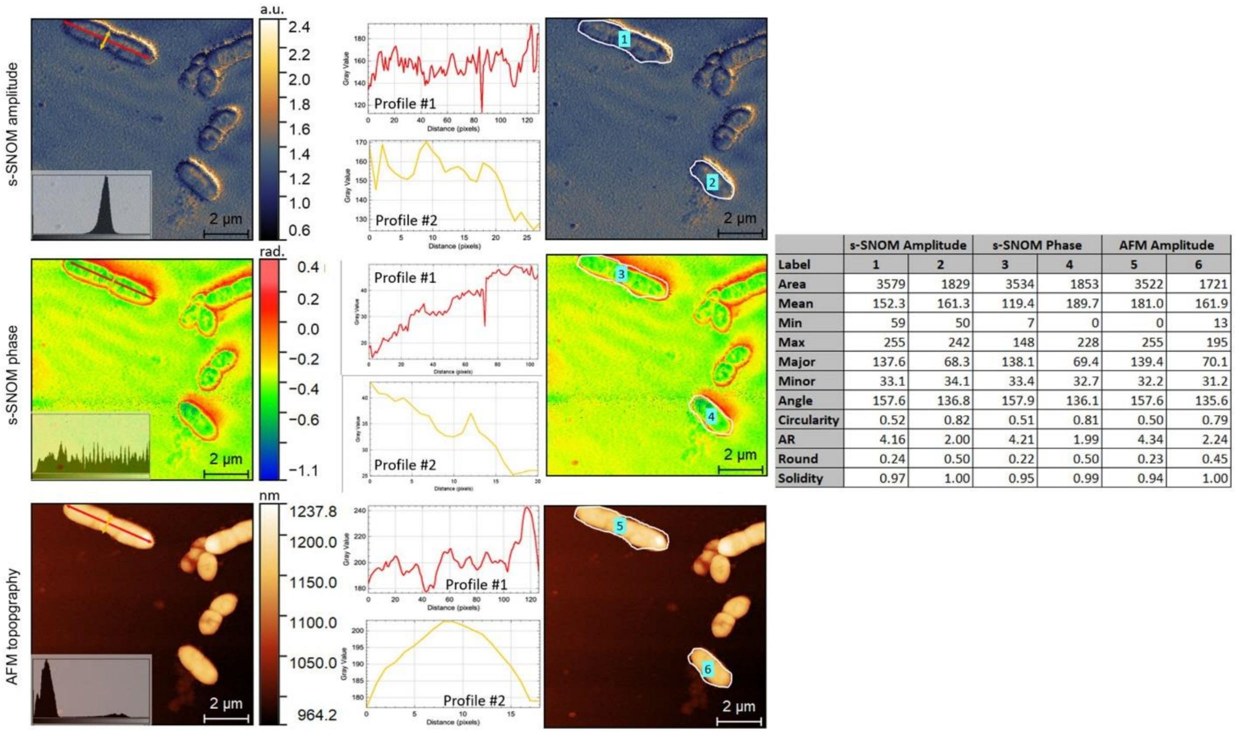

	s-SNOM Amplitude		s-SNOM Phase		AFM Amplitude	
**Label**	**1**	**2**	**3**	**4**	**5**	**6**
**Area**	3579	1829	3534	1853	3522	1721
**Mean**	152.3	161.3	119.4	189.7	181.0	161.9
**Min**	59	50	7	0	0	13
**Max**	255	242	148	228	255	195
**Major**	137.6	68.3	138.1	69.4	139.4	70.1
**Minor**	33.1	34.1	33.4	32.7	32.2	31.2
**Angle**	157.6	136.8	157.9	136.1	157.6	135.6
**Circularity**	0.52	0.82	0.51	0.81	0.50	0.79
**AR**	4.16	2.00	4.21	1.99	4.34	2.24
**Round**	0.24	0.50	0.22	0.50	0.23	0.45
**Solidity**	0.97	1.00	0.95	0.99	0.94	1.00

Fig. 5: *Example on automated feature extraction in the case of s-SNOM amplitude and phase and AFM topography images collected on Burkholderia cenocepacia ATCC BAA-245 using an in-house developed software. Once user clicks on a bacteria, ellipse fitting on the gradient image is realized and the intensity profiles along the major and minor axes of the fitted ellipse are extracted and displayed along with various features such as area and circularity of the ellipse.*

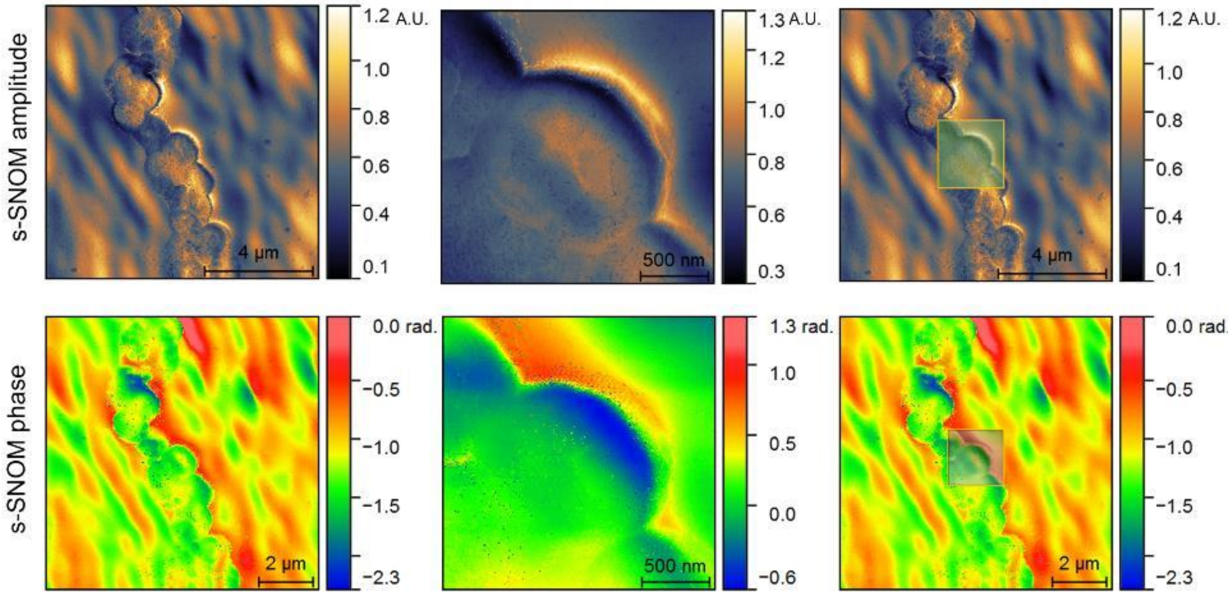

Fig. 6: *Example of cross-scale registration of s-SNOM amplitude and phase images collected on A. baumannii ATCC 17978 using an in-house developed multi-scale mutual information-based registration approach. The result of the registration is visualized on the right as a transparent overlay.*

Figure 1

Click here to access/download;Figure;Fig 1.tif

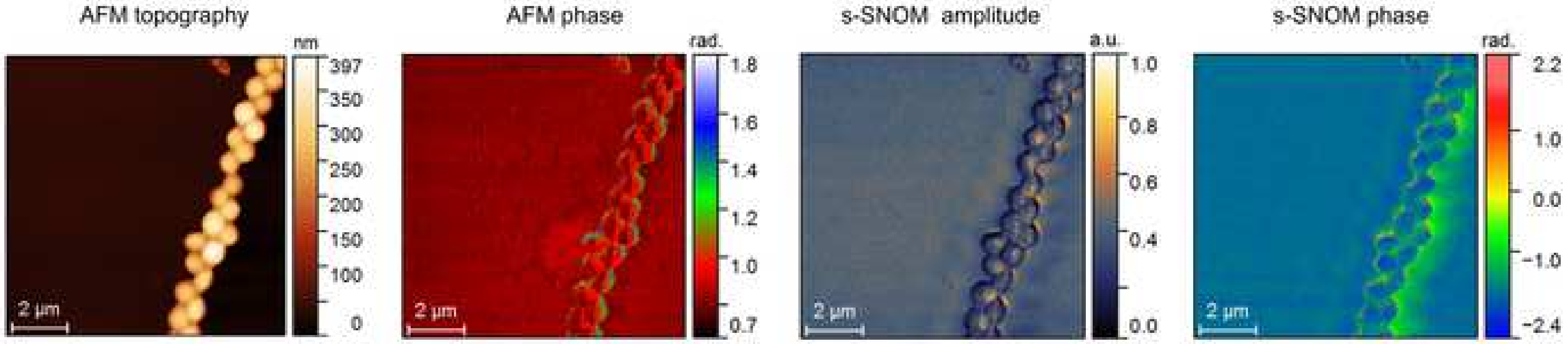

Figure 2

Click here to access/download;Figure;Fig 2.tif

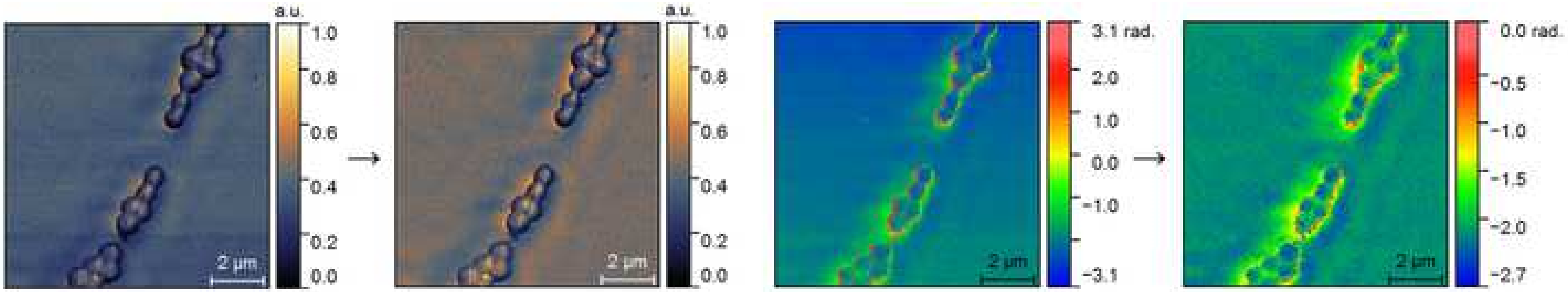

a) b)

Figure 3

Click here to access/download;Figure;Fig 3.tif

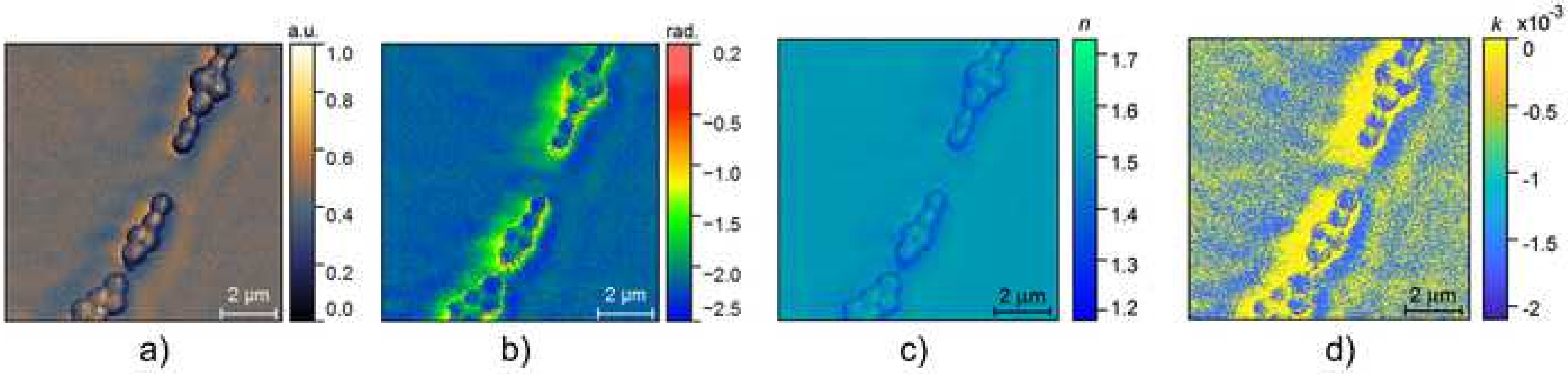

Figure 4

Click here to access/download;Figure;Fig 4.tif

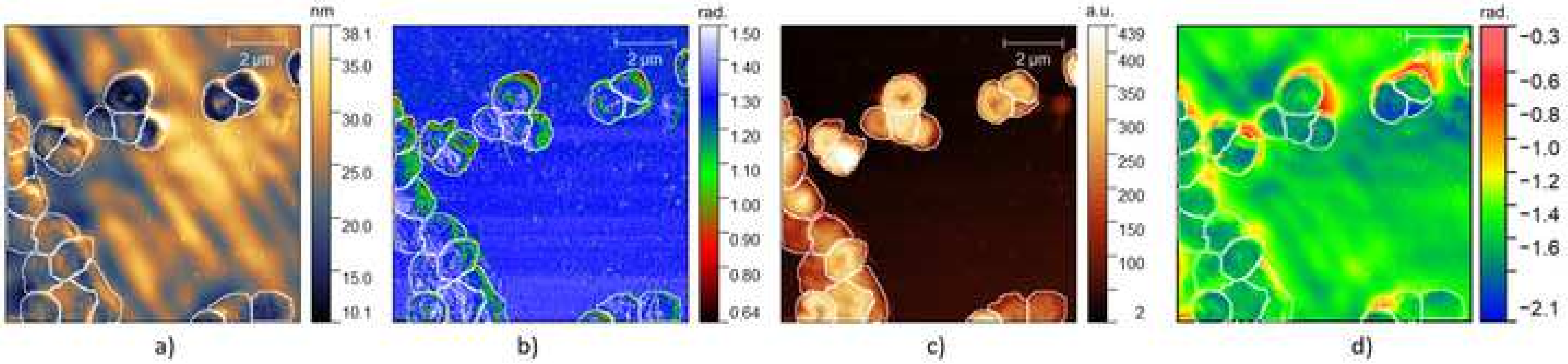

Figure 5

Click here to access/download;Figure;Fig 5.tif

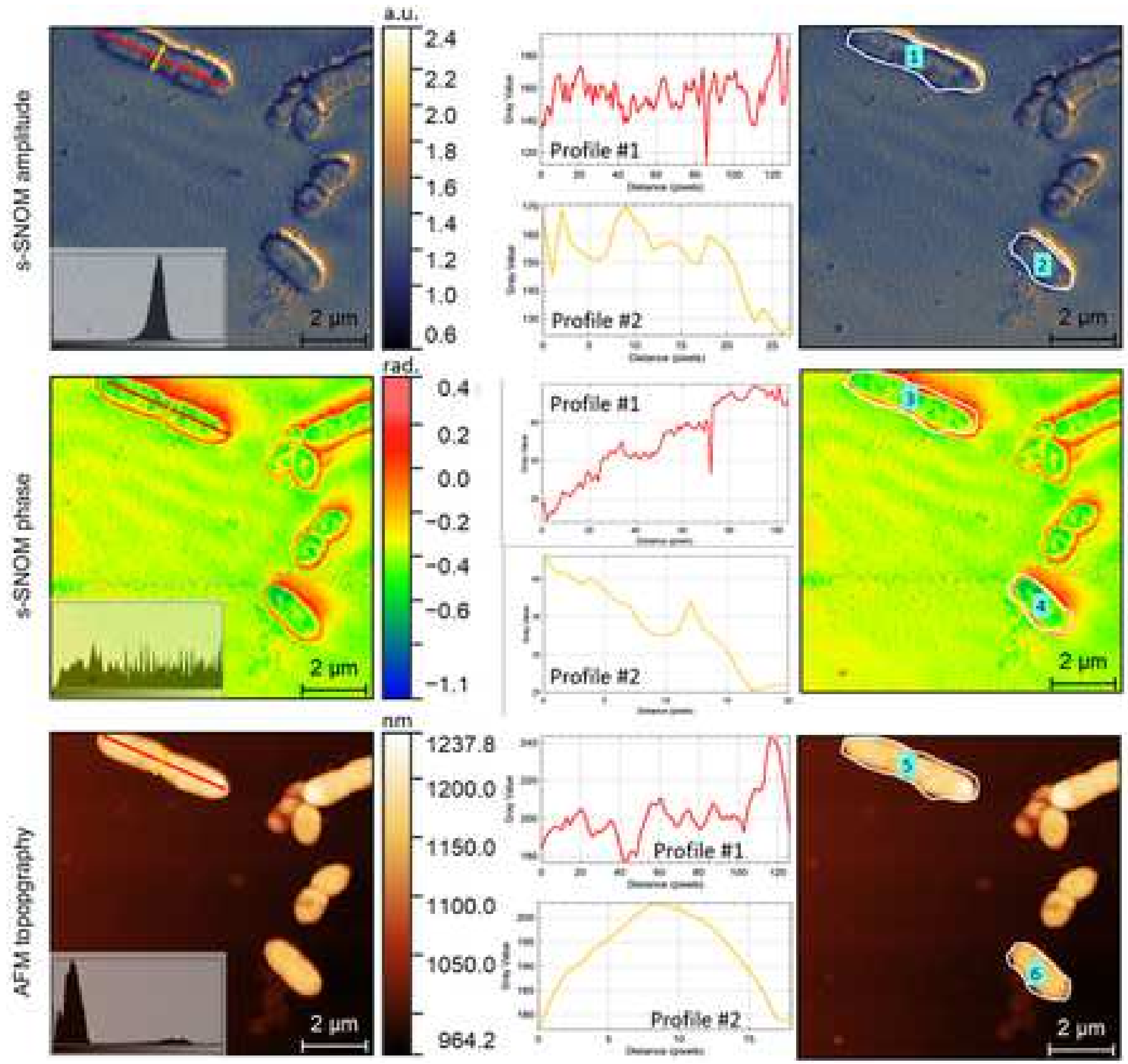

	s-SNOM Amplitude		s-SNOM Phase		AFM Amplitude	
Label	1	2	3	4	5	6
Area	3579	1829	3534	1853	3522	1721
Mean	152.3	161.3	119.4	189.7	181.0	161.9
Min	59	50	7	0	0	13
Max	255	242	148	228	255	195
Major	137.6	68.3	138.1	69.4	139.4	70.1
Minor	33.1	34.1	33.4	32.7	32.2	31.2
Angle	157.6	136.8	157.9	136.1	157.6	135.6
Circularity	0.52	0.82	0.51	0.81	0.50	0.79
AR	4.16	2.00	4.21	1.99	4.34	2.24
Round	0.24	0.50	0.22	0.50	0.23	0.45
Solidity	0.97	1.00	0.95	0.99	0.94	1.00

Figure 6

Click here to access/download;Figure;Fig 6.tif

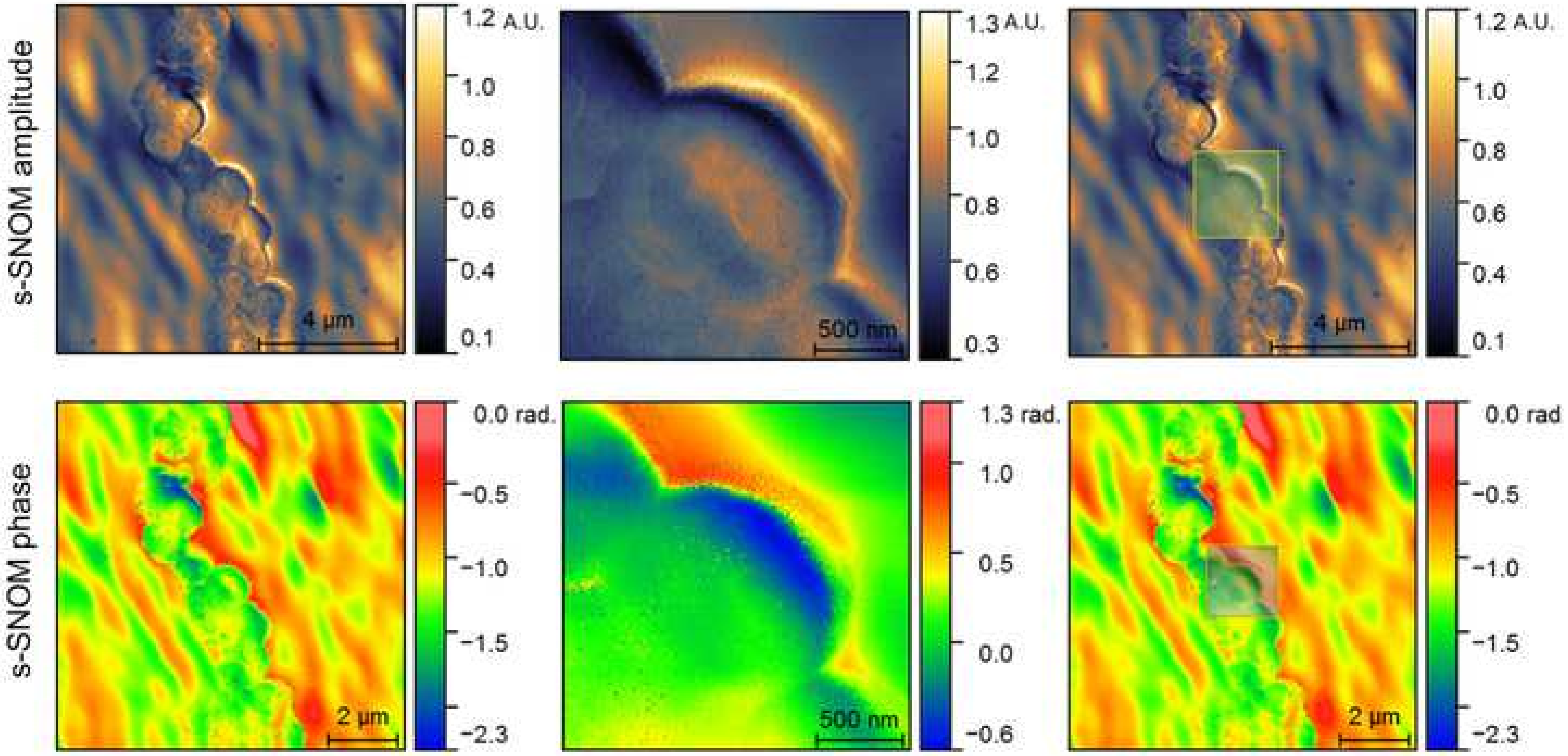

Rebuttal Letter

Click here to access/download

Supplementary Material

GigaScience SSNOMBACTER Rebuttal.pdf
