# Supplementary material for: SSNOMBACTER: A collection of scattering-type scanning near-field optical microscopy and atomic force microscopy images of bacterial cells
Source: Gigascience. 2020 Nov 24;9(11):giaa129. doi: 10.1093/gigascience/giaa129 (PMC7684706; doi:10.1093/gigascience/giaa129)
Supplement: giaa129_Response_to_Reviewer_Comments_Original_Submission [file giaa129_response_to_reviewer_comments_original_submission.pdf]

Author's Response To Reviewer Comments

Close

The rebuttal letter has been formatted in rich text format to ensure clarity, and also contains graphical elements. Due to this, we have uploaded it as a Supplementary information file. It is available for download at the end of the submitted pdf. Furthermore, the manuscript parts that have been modified to address the comments/suggestions of the Reviewers are listed in red-color font in the manuscript file.

Close
