# Supplementary material for: SSNOMBACTER: A collection of scattering-type scanning near-field optical microscopy and atomic force microscopy images of bacterial cells
Source: Gigascience. 2020 Nov 24;9(11):giaa129. doi: 10.1093/gigascience/giaa129 (PMC7684706; doi:10.1093/gigascience/giaa129)
Supplement: giaa129_Response_to_Reviewer_Comments_Revision_1 [file giaa129_response_to_reviewer_comments_revision_1.pdf]

Author's Response To Reviewer Comments

Close

We have now generated a DOI for the dataset, and addressed all pending edits suggested by Dr. Nicole Nogoy

Close
