# Supplementary material for: SSNOMBACTER: A collection of scattering-type scanning near-field optical microscopy and atomic force microscopy images of bacterial cells
Source: Gigascience. 2020 Nov 24;9(11):giaa129. doi: 10.1093/gigascience/giaa129 (PMC7684706; doi:10.1093/gigascience/giaa129)
Supplement: giaa129_Reviewer_2_Report_Original_Submission — Joachim Heberle -- 7/27/2020 Reviewed [file giaa129_reviewer_2_report_original_submission.pdf]

Reviewer Report

Title: SSNOMBACTER: A collection of scattering-type Scanning Near-Field Optical Microscopy and Atomic Force Microscopy images of bacterial cells

Version: Original Submission **Date:** 7/27/2020

Reviewer name: Joachim Heberle

Reviewer Comments to Author:

The manuscript '' SSNOMBACTER: A collection of scattering-type Scanning Near-Field Optical Miscroscopy and Atomic Force Microscopy images of bacterial cells'' by Lucidi, Cincotti, Stanciu and coworkers showcases optical nanoscale images of 15 bacterial species compiled in a database SSNOMBACTER. The work aims at increasing the popularity of sSNOM imaging in life sciences and facilitating further development of image analysis tools suited for sSNOM imaging. Furthermore, several practical examples of the usage of the database are demonstrated in the manuscript. However, a central aspect of image formation using sSNOM was omitted which is the source of the contrast, in terms of amplitude and phase. Also, a clear relation between the chosen excitation light of 1550 nm and its interaction with bacterial species at the nanoscale is missing. Thorough elaboration on this point would underline the usefulness of this very detailed database for potential users in the field of near-field imaging.
Further points to be addressed:
1) Context (1st paragraph, lines 35-57): The authors may condense this paragraph since the relevance of this information is hardly apparent for the scope of the manuscript.
2) Context (line 119): there is a recent paper showing sSNOM near-field imaging on whole cells by Meszaros, et al. in Chemical Science, 2020. Generally, the list of cited articles on sSNOM on biological specimens is incomplete. There have been several published works on lipids and lipidic domains (B. KÃ¤stner, C. Johnson, P. Hermann et al., ACS Omega, 2018), membrane proteins (S. Berwerger, D. Nguyen, E. Muller et al., JACS, 2013; I. Amenabar, S. Poly, W. Nuansing et al., Nat. Comm., 2013), individual amyloid fibrils (M. Paulite, Z. Fakhraai et al., JACS, 2011) etc.
3) Methods (line 154): Provide an explanation for using light of wavelength of 1550 nm.
4) Dataset structure (line 203): Demodulation at higher harmonics of the tip oscillation frequency is common in the recordings of near-field amplitude and phase images. I donÂ´t see any reason to have Table 1 showing abbreviations of trivial entities. What is the reason for displaying these?
5) Figure 1: x-y labels and/or scale bars as well as contrast color bars are missing.
6) Figure 2: false-color contrast bar is missing.
The image has been processed with Gwyddion which is quite a standard approach. To which respect do the authors consider the image processing novel?
7) Figure 3: the figure representation is not consistent - add scale bars; x,y axis labels, contrast bar with labels for each panel.
In b) regarding the sSNOM phase: it looks as if the minimum contrast is on the substrate, depicted in blue. If that is the case, it looks as if there is no contrast on the cell surface, but rather on the cell edges

and close surrounding to the cell (green and few red spots). Therefore, the question is what is the near-field information here?
In c) and d): while this is a nice demonstration that material properties can be extracted from sSNOM amplitude and phase, it is not clear what the physical meaning of the contrast is. Here again, the explanation of the source of the sSNOM contrast is requested.
8) Figure 4: false-color contrast bars with appropriate labels are missing.
9) Figure 5: It may be interesting to apply the feature extraction procedure on the sSNOM phase images.
10) Figure 6: provide consistent scale bars, x-y axis labels and color bars

Level of Interest

Please indicate how interesting you found the manuscript: Choose an item.

Quality of Written English

Please indicate the quality of language in the manuscript: Choose an item.

Declaration of Competing Interests

Please complete a declaration of competing interests, considering the following questions:

- Have you in the past five years received reimbursements, fees, funding, or salary from an organisation that may in any way gain or lose financially from the publication of this manuscript, either now or in the future?
- Do you hold any stocks or shares in an organisation that may in any way gain or lose financially from the publication of this manuscript, either now or in the future?
- Do you hold or are you currently applying for any patents relating to the content of the manuscript?
- Have you received reimbursements, fees, funding, or salary from an organization that holds or has applied for patents relating to the content of the manuscript?
- Do you have any other financial competing interests?
- Do you have any non-financial competing interests in relation to this paper?

If you can answer no to all of the above, write 'I declare that I have no competing interests' below. If your reply is yes to any, please give details below.

I declare that I have no competing interests

I agree to the open peer review policy of the journal. I understand that my name will be included on my report to the authors and, if the manuscript is accepted for publication, my named report including any attachments I upload will be posted on the website along with the authors' responses. I agree for my

report to be made available under an Open Access Creative Commons CC-BY license (http://creativecommons.org/licenses/by/4.0/). I understand that any comments which I do not wish to be included in my named report can be included as confidential comments to the editors, which will not be published.

Choose an item.

To further support our reviewers, we have joined with Publons, where you can gain additional credit to further highlight your hard work (see: https://publons.com/journal/530/gigascience). On publication of this paper, your review will be automatically added to Publons, you can then choose whether or not to claim your Publons credit. I understand this statement.

Yes Choose an item.
